# Association Between Asthma and All-Cause Mortality and Cardiovascular Disease Morbidity and Mortality: A Meta-Analysis of Cohort Studies

**DOI:** 10.3389/fcvm.2022.861798

**Published:** 2022-03-17

**Authors:** Bin Zhang, Zhi-Fei Li, Zhuo-Yu An, Li Zhang, Jing-Yi Wang, Meng-Di Hao, Yi-Jing Jin, Dong Li, An-Jian Song, Qiang Ren, Wen-Biao Chen

**Affiliations:** ^1^Department of Cardiovascular Disease and Clinical Experimental Center, Jiangmen Central Hospital, Jiangmen, China; ^2^Aerospace Center Hospital, Beijing, China; ^3^Peking University People's Hospital, Beijing, China; ^4^Peking University First Hospital, Beijing, China; ^5^Department of Intensive Care Unit and Clinical Experimental Center, Jiangmen Central Hospital, Jiangmen, China; ^6^Department of Respiratory Medicine, People's Hospital of Longhua, The Affiliated Hospital of Southern Medical University, Shenzhen, China

**Keywords:** asthma, cardiovascular disease, cardiovascular disease morbidity, cardiovascular disease mortality, all-cause mortality

## Abstract

**Background:**

Asthma and cardiovascular disease (CVD) share many risk factors. Previous meta-analyses indicated that asthma is associated with an increased risk of CVD and all-cause mortality, but these studies were limited by unstandardized search strategies and the number of articles included.

**Objective:**

We sought to systematically synthesize evidence investigating the impact of asthma on all-cause mortality and CVD morbidity and mortality.

**Methods:**

We searched in PubMed and EMBASE for observational cohort studies (inception dates to November 10, 2021) that had both asthma groups and control groups. We also manually searched the reference lists of correlative articles to include other eligible studies. Data for associations between asthma and all-cause mortality and CVD morbidity and mortality were needed.

**Results:**

We summarized the findings from 30 cohort studies comprising 4,157,823 participants. Asthma patients had increased CVD morbidity [relative risk (RR) = 1.28, 95% confidence interval (CI) = 1.16–1.40] and increased CVD mortality (RR = 1.25, 95% CI = 1.14–1.38). Asthma patients also had increased risk of all-cause mortality (RR = 1.38, 95% CI = 1.07–1.77). In subgroup analyses, female asthma patients had a higher risk of CVD morbidity and all-cause mortality than male asthma patients, and late-onset asthma patients had a higher risk of CVD morbidity than early-onset asthma patients.

**Conclusion:**

Asthma patients have increased risk of all-cause mortality and CVD morbidity and mortality. This information reminds clinicians to be aware of the risk of CVD and all-cause mortality in asthma patients.

**Systematic Review Registration:**

http://www.crd.york.ac.uk/PROSPERO/, PROSPERO, identifier: CRD 42021290082.

## Introduction

Asthma is the most common inflammatory chronic non-infectious pulmonary disease in adults and children, and is estimated to affect 334 million people worldwide (1). Asthma is more common in developed countries and imposes a serious health and economic burden worldwide (2, 3). Chronic airway inflammation and airway hyperresponsiveness are the main pathophysiological features of asthma, which manifests as progressive airway remodeling (4, 5). The pathogenesis of asthma involves immune-inflammatory mediators and neuromodulator mechanisms (6). Asthma and cardiovascular disease (CVD) share many risk factors, such as inflammation (7). Patients with asthma suffer from a chronic inflammatory state, and asthma patients have been shown to have persistently elevated levels of inflammatory factors, such as fibrinogen and tumor necrosis factor-1 (8). In addition, poor lung function and eosinophilia, which are characteristic of asthma, have been shown to be predictors of cardiovascular mortality (9–11).

As asthma is very common and the consequences of CVD are critical, understanding their relationship will provide useful information to clinicians treating patients with asthma. Moreover, the long-term prognosis of asthma patients is one of the crucial questions that need to be addressed in this field. Understanding the impact of asthma on long-term mortality and cardiovascular risk and mortality is important for the development of clinical practice guidelines.

Previous meta-analyses have indicated that asthma is associated with an increased risk of CVD and all-cause mortality, but these studies were limited by unstandardized search strategies and a failure to include recent, updated articles (12–14). To overcome these limitations and provide reliable conclusions, we conducted a systematic evaluation and meta-analysis to investigate the impact of asthma on all-cause mortality and CVD morbidity and mortality.

## Materials and Methods

We conducted this meta-analysis following Meta-Analysis of Observational Studies in Epidemiology (MOOSE) guidelines (15) and Preferred Reporting Items for Systematic Reviews and Meta-Analyses (PRISMA) (Supplementary Table 3). The meta-analysis was registered with the International Prospective Register of Systematic Reviews (PROSPERO identifier: CRD 42021290082).

### Literature Search

We searched PubMed and Embase for cohort studies from inception to November 10, 2021, without language restrictions. See the Supplementary File for search details (Supplementary Table 1). We also included other eligible studies by manually searching the reference lists of former similar meta-analyses and relevant studies.

### Eligibility Criteria

We included only observational cohort studies without publication time restriction. Both retrospective and prospective cohort studies were included if they had both asthma groups and control groups. Theoretically, in included studies, the difference between asthma groups and control groups should only be whether they had asthma or not. We excluded studies which only included specific asthma patients such as having ICS treatment or overlapping chronic obstructive pulmonary disease. To assess comparability between the asthma groups and the control groups, we included studies that provided baseline characteristics of the population. Outcome measures on the association between asthma and all-cause mortality or CVD morbidity and mortality were necessary. The total number of subjects in the studies must be >1,000 to reduce bias.

### Data Extraction

A data collection table was made in advance. The correlative data were extracted by two investigators (Z-FL and Z-YA) independently. When there was disagreement, a third person (BZ) would assist in the discussion and make the final decisions. Any collected data were aggregate, and no individual-level data were included.

### Quality Assessment

The risk of bias and quality of studies were evaluated by the Newcastle-Ottawa Scale (NOS) for cohort studies. The NOS ranges from 0 to 9 points. When the NOS is <6 points, the study would be regarded low quality; 6 or 7 points, the study would be regarded medium quality; and more than 7 points, the study would be regarded high quality. Two authors (LZ and J-YW) finished the quality evaluation. When there was disagreement, a third person (BZ) would assist in the discussion and make the final decisions.

### Outcomes of Interest

Data for associations between asthma and all-cause mortality and CVD morbidity and mortality were needed, such as relative risk (RR), hazard ratios (HRs), or number of CVD events (morbidity, mortality) or all-cause deaths in the asthma and control groups.

### Statistical Analysis

At first, we measured both adjusted and unadjusted RRs/HRs. Then, we pooled the adjusted RRs that were initially provided in the studies or estimated by the HRs. I^2^ statistic were used to evaluate the heterogeneity of included studies. when I^2^ >50%, significant heterogeneity was considered existing (16) and the random-effects model was used. Funnel plots were used to evaluate publication bias. RevMan 5.4.1 (Nordic Cochrane Center, Cochrane Collaboration) were used to conduct all calculations and draw forest plots to show the results. Stata version 14.0 (Stata Corp., College Station, TX, USA) were used for sensitivity analysis.

## Results

**Figure 1:**
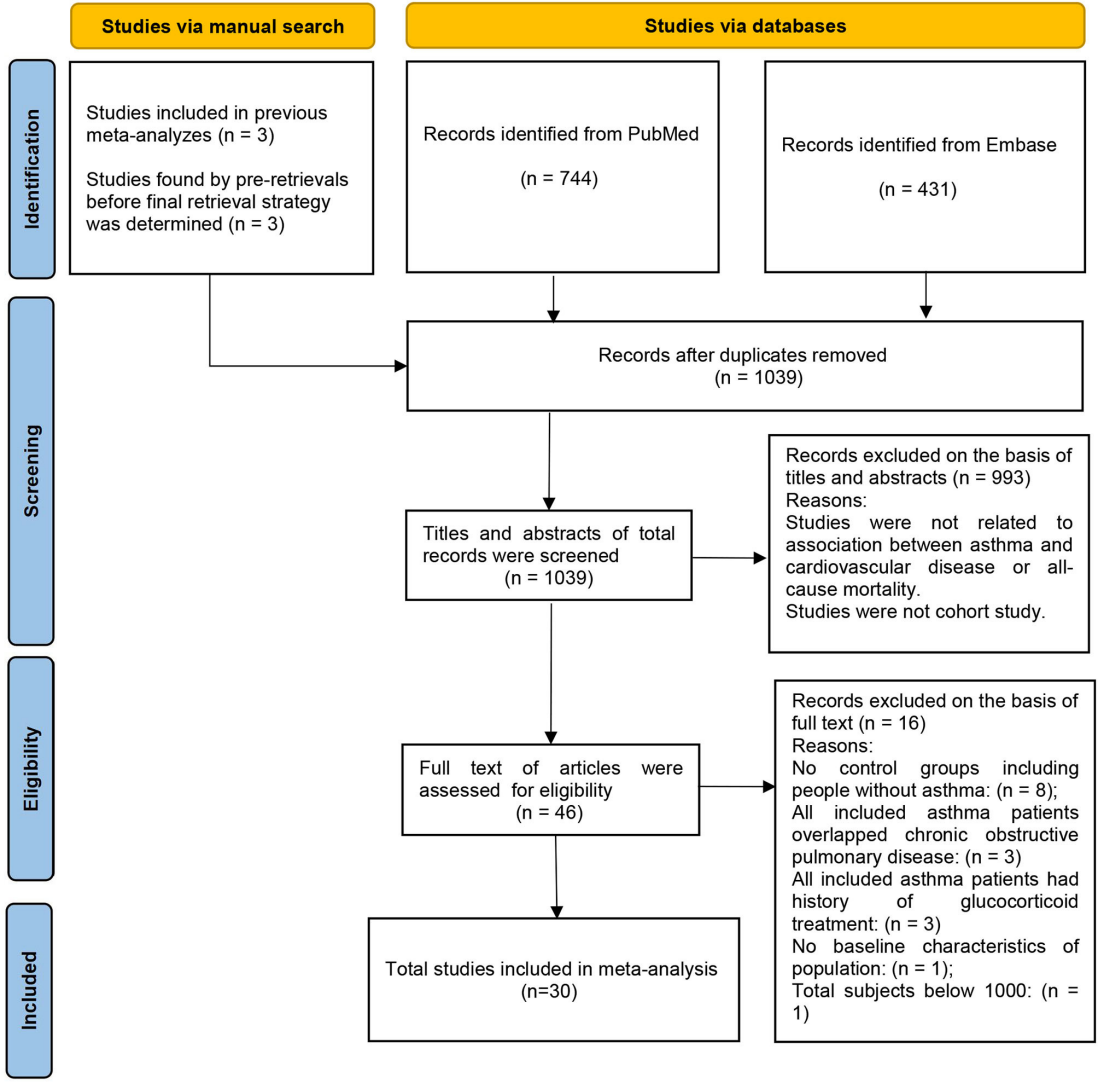
Flow chart of article selection process.

Thousand thirty nine studies were yielded by the electronic search of Pubmed and Embase databases and manual search. After applying the inclusion and exclusion criteria, 30 studies (17–46) were included in the meta-analysis, including 5 studies (42–46) assessed from the manual search and 25 studies (17–41) from the electronic database search (Figure 1).

### Description of Studies

All 30 studies were observational cohort studies, including 25 prospective cohort studies and 5 retrospective cohort studies. We collected the baseline characteristics of each study (Table 1). These studies comprised 4,157,823 participants. Thirteen studies were done in Europe, 12 in North America, 3 in Oceania, and 2 in Asia. In our quality assessment used the NOS (Supplementary Table 2), 17 studies were regarded as high quality, 13 as medium quality, and none as low quality. Nine studies included male and female subgroups. Three studies included early and late-onset asthma subgroups.

**Table 1:** Baseline characteristics of participants assessed in the studies included in the meta-analysis.

**Study authors, year of publication, location**	**Total**								**Define of cardiovascular diseases**	**Confounding factors adjusted for in the analyses**	**NOS score**
	**Population**	**Participant number**	**Study type**	**Follow-up, mean or (range)**	**Age, range or (mean** **±SD)**	**Gender (female, %)**	**diagnosis criteria of asthma**	**Comorbidities**, ***n*** **(%)**			
Ingebri et al. (42), 2020, Denmark	community	44,177	prospective	5.7	57.9 ± 11.0	61%	Self-reported	DM:2.8%	CHD, HF	Age, gender, family history of CVD, smoking, BMI, HTN, DM, TC levels and physical activity in leisure time	8
Bellia et al. (44), 2007, Italy	hospital	1,233	prospective	4.8	>65	53%	Doctor diagnosis	CVD:15.7% CBVD:6.0% DM:12.2%	NA	Age, gender, smoking habit, physical and cognitive, mood status, FEV1%, FVC%, FEV1/FVC, BMI, Charlson index of comorbidity, SGRQ, inhaled CS or β_2_adrenergic drugs	8
Liss et al. (45), 2000, Canada	hospital	2,400	prospective	8	17–65	40%	Doctor diagnosis	NA	IHD	Period of birth, time period of accident, sex	6
Çolak et al. (46), 2015, Denmark	community	42,489	prospective	4.5	20-100	59%	Self-reported	NA	IHD	NA	7
Lange et al. (19),1996,Denmark	community	13,540	prospective	17	>20	55%	Self-reported	NA	ICD-8 (390-459)	Age, asthma, smoking habits, inhalation habits, length of school education, chronic mucus hypersecretion	8
Ali et al. (29), 2013, Denmark	community	2,150	prospective	25.6	>15	60%	Doctor diagnosis	NA	ICD-10 (DI20)	Age, FEV1%	8
Dantzer et al. (21), 2001, France	community	2,348	prospective	8	>65	59%	Self-reported	NA	NA	Sex, educational level, marital status, cognitive impairment, smoking and a previous history of IHD, infarcts and cardiovascular accident.	7
Diaz-Guzman et al. (26), 2011, America	community	15,203	prospective	18	>25	53%	Self-reported	NA	NA	Age, sex, race/ethnicity, smoking status, education level, BMI, reported respiratory disease and disease stage	7
Huovinen et al. (20), 1997, Finland	community	30,249	prospective	16	>18	51%	Doctor diagnosis	NA	NA	Age, smoking, social class, pets, dogs, CB, dyspnea, hay fever	9
Markowe et al. (17), 1987, England and Wales	community	5,094	prospective	8.4	25-64	55%	Doctor diagnosis	NA	ICD-8 (390-459)	NA	8
Ulrik and Frederiksen (18), 1995, Denmark	outpatient	2,150	prospective	8.6	>15	60%	Doctor diagnosis	NA	ICD-8(410)	NA	8
Vandentorren et al. (22), 2003, France	community	1,4267	retrospective	25	25-59	52%	Self-reported	NA	NA	Age, sex, educational level, smoking habits, occupational exposure, FEV1	8
Chung et al. (30), 2014, China	community	38,840	prospective	1-−20	≥18	54%	Doctor diagnosis	HTN:32.5% DM:11.6% HPL:16.6% Stroke:8.7% HF:2.4% COPD:27.1%	ACS	Age, sex, comorbidities of HTN, DM, HPL, stroke, HF and COPD	8
Iribarren et al. (23), 2004, America	community	151,620	retrospective	27	15-92	54%	Doctor diagnosis	HTN:18.5% DM:18.0%	CHD	Age, race/ethnicity, education level, smoking Status, alcohol consumption, BMI, serum TC, white blood cell count, HTN, DM, parental history of CHD, and occupational exposures.	7
Iribarren et al. (27), 2012, America	community	407,190	prospective	5.2-6.3	≥18	52%	Doctor diagnosis	HTN:7.2% DM:5.6% HPL:6.1%	CHD	DM, HTN, HPL, BMI, and smoking status, prior history of any allergy	7
Onufrak et al. (25), 2008, America	community	14,567	prospective	12-14	45-64	54%	Self-reported	DM:10.9% HTN:33.5% CB:8.4%	CHD	Age, BMI, black race, DM, HTN, education level, LDL-C, HDL-C, physical activity	8
Schanen et al. (24), 2005, America	community	1,3797	prospective	14	45-64	57%	Self-reported	HTN:23.0% DM:10.4%	CHD	Age, sex, race/center, HDL-C, LDL-C, systolic blood pressure, HTN medication use, smoking status, pack years, W/H ratio, DM, sport score	7
Yun et al. (28), 2012, America	community	7,176	retrospective	Asthma:2.9-13.2, control:1.9-12	15.1 ± 20.5	43%	Doctor diagnosis	NA	CHD	Race/ethnicity (white vs. non-white)	7
Ng, et al. (43), 2020, Australia	school	4,430	retrospective	16	11.8 ± 2.9	50%	Self-reported	NA	NA	Birth year, age at child survey, and gender	7
Eftekhari, et a. (33), 2016, Australia	community	10,413	prospective	11.9	73-78	100%	Self-reported	HTN:33.0%, DM:7.3%, heart disease:13.1%, thrombosis:1.5%, stroke:2.7%	NA	Age, demographic factors, thrombosis, osteoporosis, low iron level, HTN, DM, breast cancer, stroke, other major illness, residential area, social support	8
Strand, et al. (36), 2018, China	community	446,346	prospective	17	40.0 ± 13.5	51%	Self-reported	HTN:18.2%, DM:5.0%, heart disease/heart surgery/use of heart drugs:3.6%, stroke:0.5%	CHD, stroke	Age, education, marital status, smoking, alcohol consumption, physical activity, DM, HTN, BMI, TC, TG, history of heart disease/heart surgery/use of heart drugs and history of stroke.	7
Lemmetyinen et al. (35), 2018, Finland	community	2,941	prospective	15.6	>30	50%	Doctor diagnosis and self-reported	Allergic rhinitis:36.2%, Allergic conjunctivitis:27.6%, Atopic dermatitis:27.6%	ICD-10 (I05-I99)	Age, sex, pack-years, education, and BMI	8
Cepelis et al. (37), 2019, Norway	community	57,104	prospective	17.2	>20	54%	Self-reported	DM:2.2%, HTN:37.5%	AMI	Age, sex, BMI, smoking status, alcohol use, physical activity, education level, TC/HDL ratio, HTN, DM	8
Caffrey Osvald et al. (38), 2020, Sweden	community	277,5430	retrospective	13.7	1-27	49%	Doctor diagnosis	NA	NA	Sex, prematurity, maternal age at delivery, maternal smoking during pregnancy, maternal country of birth, parental income, parental education	8
He et al. (40), 2021, America	community	37,015	prospective	7.5	>20	52%	Self-reported	DM:8.8%, CHD:3.3%, stroke:2.7%, cancer:9.3%, arthritis:24.1%	NA	Age, sex, race, education, income, smoking status, BMI, DM, arthritis, CHD, stroke, cancer	7
Huang et al. (31), 2015, America	community	1,830	prospective	40	21-80	56%	Doctor diagnosis	NA	NA	Age, sex, education, BMI, smoking status and pack-years	8
Pollevick et al. (41), 2021, America	community	3,612	prospective	35	17-77	53%	Doctor diagnosis	DM:2.4%	MI, angina, coronary insufficiency, stroke transient ischemic attack, HF	Age, sex, HDL-C, TC, currently taking anti-HTN medications, systolic blood pressure, DM, currently smoking, obesity, and education	8
Ng et al. (39), 2021, Australia	school	2,153	prospective	20.2	32.4	50%	Doctor diagnosis and self-reported	DM:2.5%, history of hay fever:33.0%	ICD-9 (390-459), ICD-10 (I00-99, G45)	Birth year, age at child survey, and gender	7
Tattersall et al. (34), 2016, America	employees	1,267	prospective	13.9	47.3 ± 8.0	44%	self-reported	HTN:29.4%, DM:3.0%	coronary death, MI, angina, stroke, coronary revascularization, HF	Age, sex, smoking status, DM, lipid medications, HTN, BMI	7
Tattersall et al. (32), 2015, America	community	6,792	prospective	9.1	62.2 ± 10.3	52%	self-reported	DM:12.6%	CHD, stroke, angina	Age, race, sex, TC, HDL-C, systolic blood pressure, smoking, DM, anti-HTN and lipid-lowering medication use at baseline, BMI, family history of CVD, income	8

### Association Between Asthma and All-Cause Mortality

**Figure 2:**
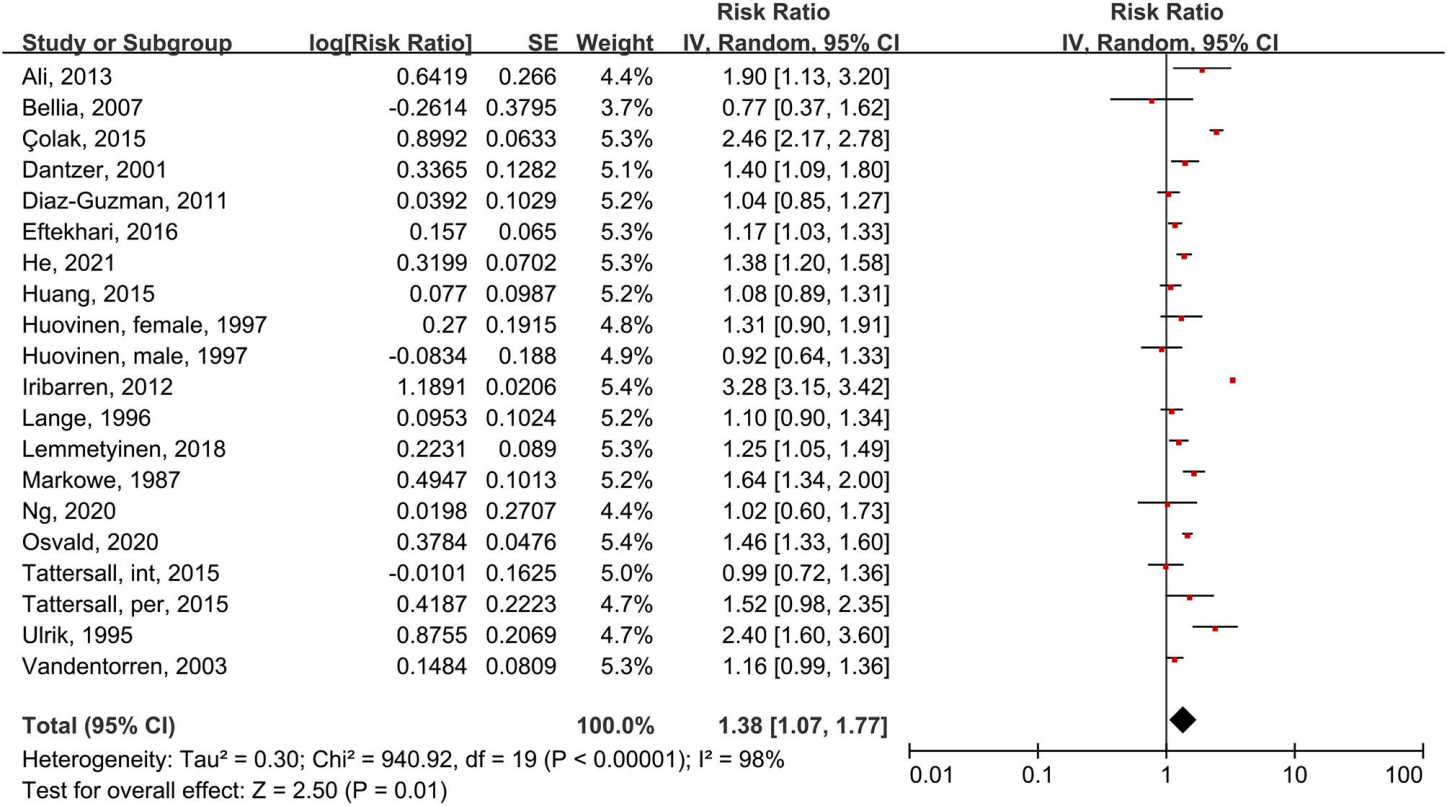
Forest plot shows association between asthma and all-cause mortality. SE, standard error; IV, Inverse Variance method; df, degrees of freedom; int, intermittent; per, persistent.

Using the random-effects model, we analyzed 18 studies that included 20 subgroups (Figure 2). Asthma patients had a 38% increased risk of death compared to people without asthma (RR = 1.38, 95% CI = 1.07–1.77, p = 0.01, I^2^ = 98%, *p* < 0.00001). Though considerable heterogeneity existed, most studies or subgroups (16 in 20) indicated an increased risk of mortality in asthma patients. We also found high heterogeneity, mainly because a large-scale study (27) conducted by Iribarren et al. in 2012 reported an outlier HR (3.28, 95% CI = 3.15–3.41).

**Figure 3:**
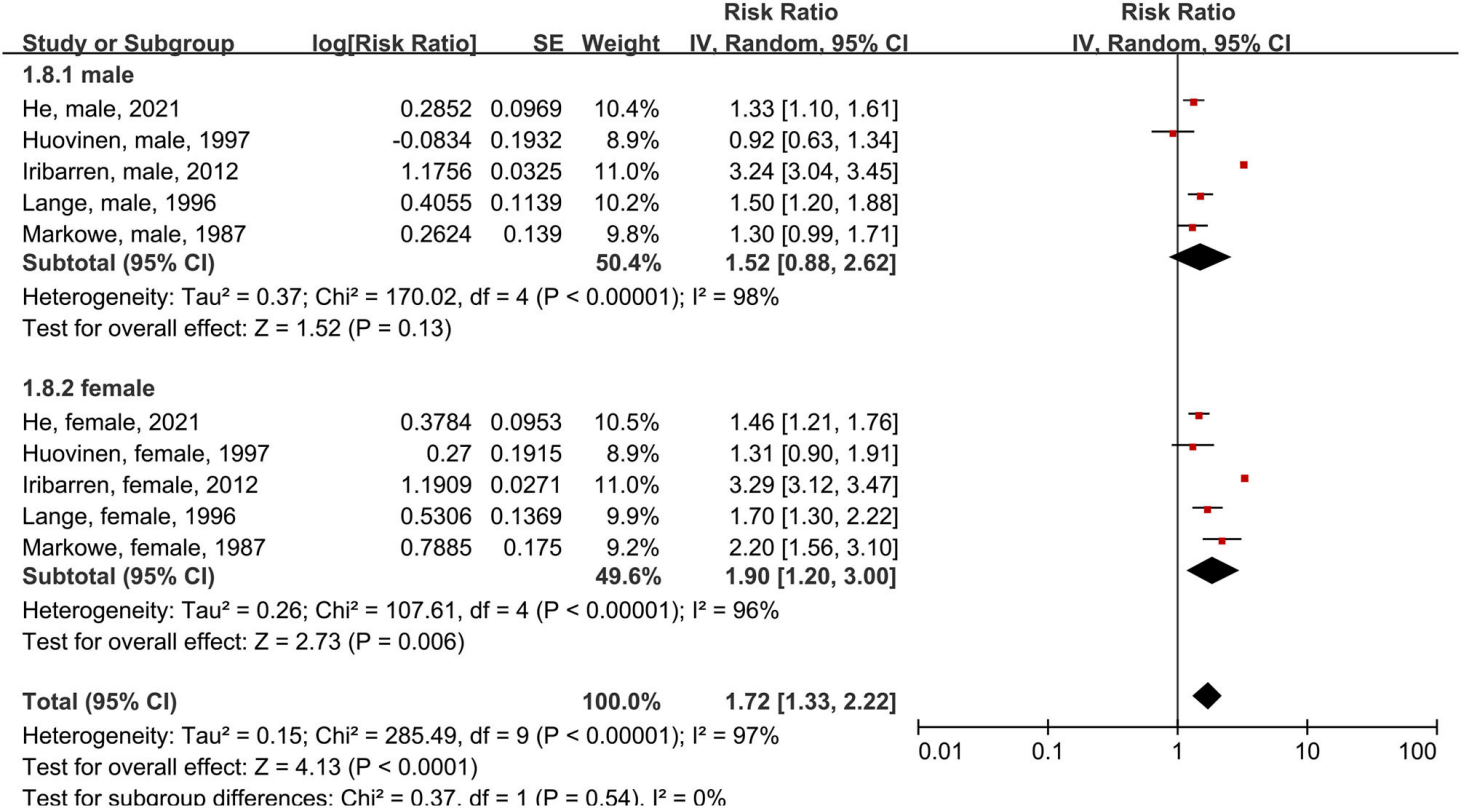
Forest plot shows association between asthma and all-cause mortality in male and female patients. SE, standard error; IV, Inverse Variance method; df, degrees of freedom.

Five studies were included in the subgroup analysis of male and female patients using the random-effects model (Figure 3). In male asthma patients, risk of all-cause mortality increased 52% compared to people without asthma, though this difference was not significant (RR = 1.52, 95% CI = 0.88–2.62, *p* = 0.13; I^2^ = 98%, *p* < 0.00001). In female patients, risk of all-cause mortality increased 90% compared to people without asthma (RR = 1.90, 95% CI = 1.20–3.00, *p* = 0.006; I^2^ = 96%, *p* < 0.00001).

### Association Between Asthma and CVD Morbidity

**Figure 4:**
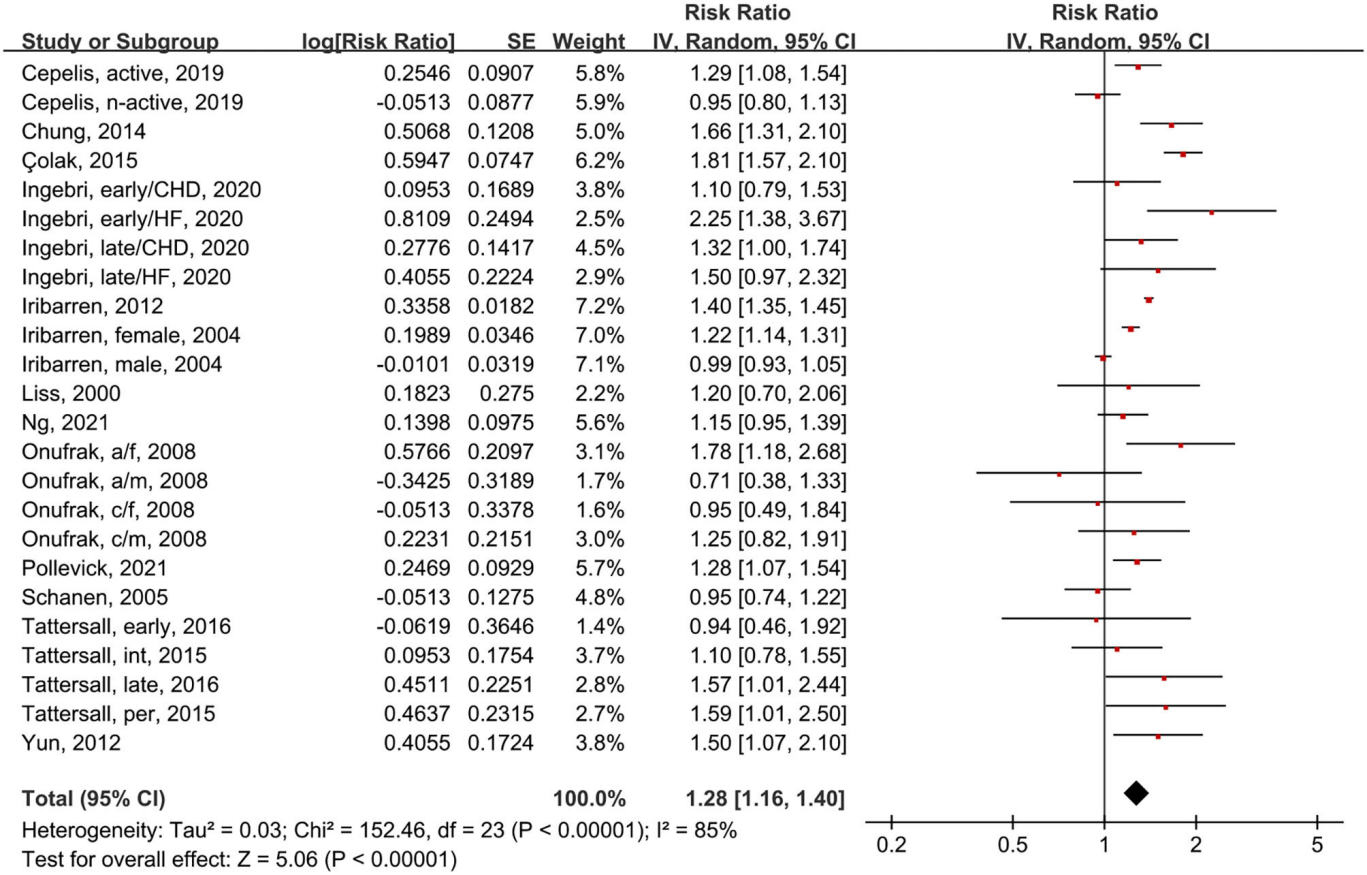
Forest plot shows association between asthma and CVD morbidity. SE, standard error; IV, Inverse Variance method; df, degrees of freedom; HF, heart failure; CHD, coronary heart disease; a/f, adult female; a/m, adult male; c/f, child female; c/m, child male; int, intermittent; per, persistent.

We analyzed 14 studies with a total of 24 subgroups (Figure 4). The random-effects model showed that asthma patients had a 32% increased risk of CVD compared to people without asthma (RR = 1.28, 95% CI = 1.16–1.40, *p* < 0.00001; I^2^ = 85%, *p* < 0.00001). Thus, asthmatic status increases the risk of CVD morbidity.

**Figure 5:**
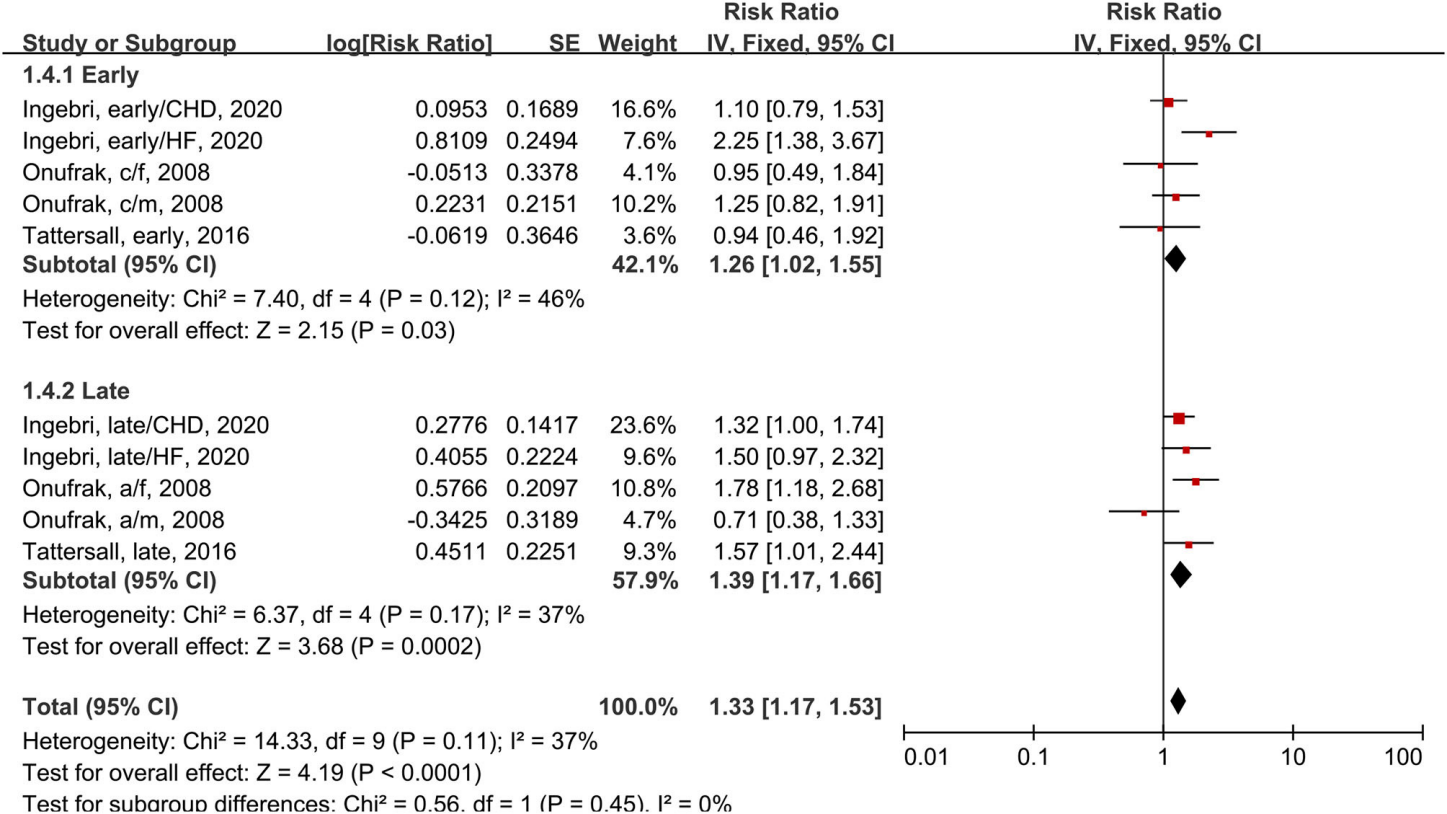
Forest plot shows association between asthma and CVD morbidity in early and late-onset asthma patients. SE, standard error; IV, Inverse Variance method; df, degrees of freedom; HF, heart failure; CHD, coronary heart disease; a/f, adult female; a/m, adult male; c/f, child female; c/m, child male.

In the subgroup analysis of early and late-onset asthma using the fixed-effects model, three studies had a total of five subgroups (Figure 5). One study (34) defined early-onset as asthma onset prior to 18 years and late-onset as asthma onset at 18 years or older. The other two studies (25, 42) applied the cutoffs of 40 years and 21 years. Early-onset asthma patients has a 26% increased risk of CVD compared to people without asthma (RR = 1.26, 95% CI = 1.02–1.55, *p* = 0.03; I^2^ = 46%, *p* = 0.12). Late-onset asthma patients had a 39% increased risk of CVD compared to people without asthma (RR = 1.39, 95% CI = 1.17–1.66, *p* = 0.0002; I^2^ = 37%, *p* = 0.17). This subgroup analysis reduced the heterogeneity of studies.

**Figure 6:**
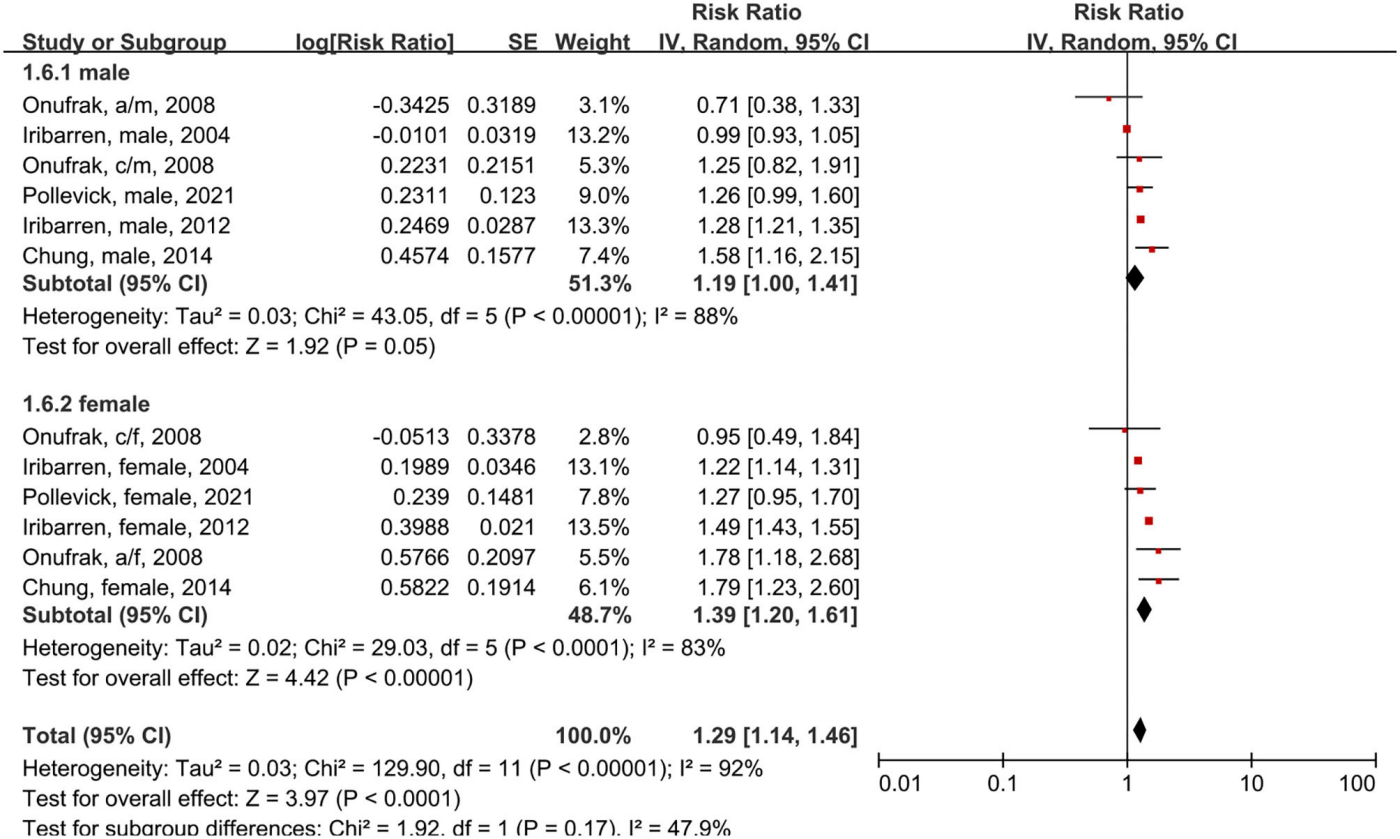
Forest plot shows association between asthma and CVD morbidity in male and female asthma patients. SE, standard error; IV, Inverse Variance method; df, degrees of freedom; a/f, adult female; a/m, adult male; c/f, child female; c/m, child male.

In the subgroup analysis of male and female patients using the random-effects model, five studies had a total of six subgroups (Figure 6). Male asthma patients had an 18% higher risk of CVD morbidity compared to people without asthma (RR = 1.19, 95% CI = 1.00–1.41, p = 0.05; I^2^ = 88%, *p* < 0.00001). Female asthma patients had a 37% higher risk of CVD compared to people without asthma (RR = 1.39, 95% CI = 1.20–1.61, *p* < 0.00001; I^2^ = 83%, *p* < 0.0001).

### Association Between Asthma and CVD Mortality

**Figure 7:**
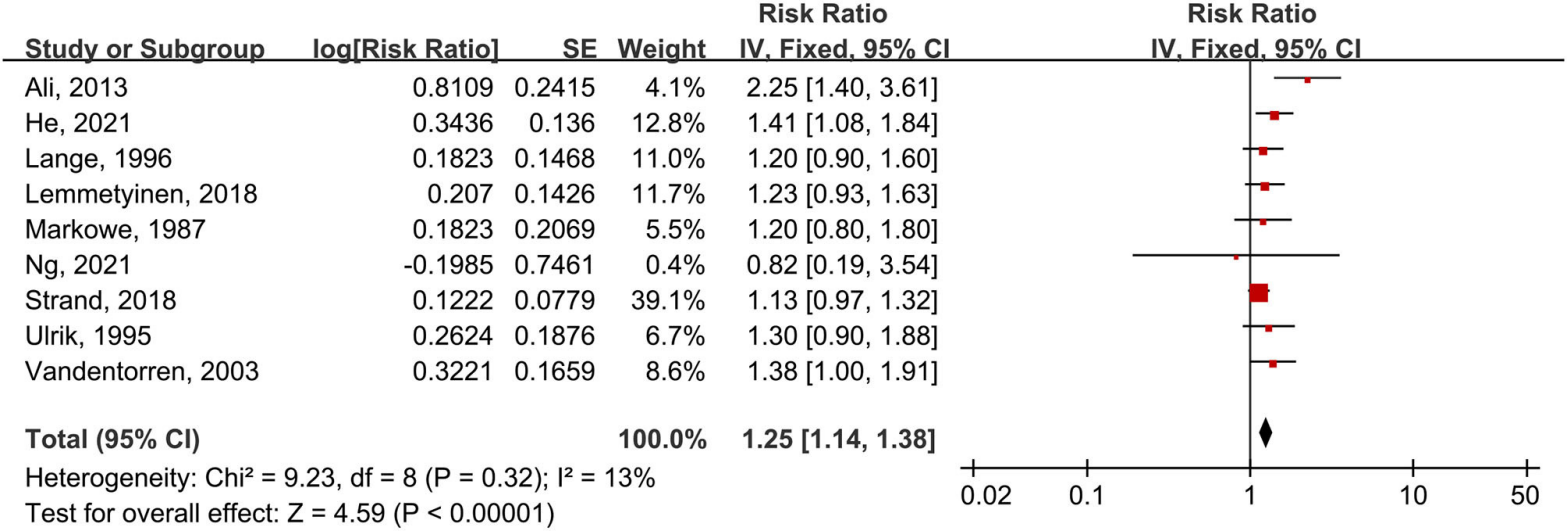
Forest plot shows association between asthma and CVD mortality. SE, standard error; IV, Inverse Variance method; df: degrees of freedom.

Nine studies provided the outcomes of CVD mortality in asthma patients. These studies were summarized for the first time (Figure 7). Data calculated by the fixed-effects model showed that asthma patients have a 25% increased risk of CVD mortality compared to people without asthma (RR = 1.25, 95% CI = 1.14–1.38, *p* < 0.00001). There was no considerable heterogeneity between these studies (I^2^ = 13%, *p* = 0.32). Eight of nine studies yielded an increased risk of CVD mortality in asthma patients. Six of nine studies were regarded as high quality.

### Sensitivity Analysis

We conducted sensitivity analysis to examine the robustness of the meta-analyses by omitting each study in turn and recalculating the pooled effect estimates (Supplementary Figures 4–12). In male asthma patients, one study (27) altered the significantly increased risk of all-cause mortality to no significant difference. However, this study did not change the estimate significantly. Two subgroups of two studies (25, 42) decreased the estimate of CVD morbidity in early-onset asthma patients significantly (Supplementary Figure 9). However, this may due to that two studies did not provide the overall estimate, and we input the grouping estimate (male, female and CHD, HF) provided by the studies. Overall mortality analysis has a I square of 98% (highly heterogenous), and we conducted sensitivity analysis by omitting two outliers [Colak et al. (46) and Iribarren et al. (27)] and result still robust (Supplementary Figure 13).

### Publication Bias

Publication bias was evaluated using funnel plots, and no clear asymmetry was found by visual inspection (Supplementary Figures 1–3). We noted that one study (27) had relatively high loss to follow-up (24% of asthma subjects, 10% of control subjects) and had a significant outlier outcome (Supplementary Figure 3). Overall, no significant publication bias was found among the included observational cohort studies.

## Discussion

In this systematic review, we found that asthma patients have a significantly increased risk of CVD morbidity and mortality and all-cause mortality. Male asthma patients had no significant increased risk of all-cause mortality, and lower risk of CVD morbidity and all-cause mortality than female asthma patients. In addition, early-onset asthma patients had a lower risk of CVD morbidity than late-onset asthma patients.

### Association Between Asthma and All-Cause Mortality

Finding that asthma patients have an increased risk of all-cause mortality confirms the results of a previous meta-analysis (14). However, a large-scale study (27) reporting a considerably high risk of all-cause mortality is the main reason for heterogeneity between studies and the most conspicuous outlier in the funnel diagram. We considered two main reasons for this outlier outcome. First, high loss to follow-up can bias the results. Second, the inclusion criteria for asthma patients were one hospitalization, two outpatient visits, or two emergency department visits for asthma within 12 months, whereas most of the other studies based the inclusion criteria on questionnaires about self-reported asthma history. Thus, all of the asthma patients in that study had active and more severe asthma and, therefore, higher CVD morbidity and mortality risk than non-active asthma patients (37, 40).

Though asthma patients have an increased risk of all-cause mortality, only a small number of asthma patients die directly from asthma itself (35). Thus, an increased risk of death in asthma patients is mainly due to comorbidities. Asthma patients always have chronic inflammation and airway limitations, which can elevate the risk of chronic obstructive pulmonary disease (COPD) and type 2 diabetes (47). In addition, asthma medication can cause comorbidities. Inhaled corticosteroids may be related to osteoporosis, increased fracture risk, and pneumonia (48). Inhaled β2-agonist can increase the risk of CVD (49). Furthermore, suboptimal asthma control can limit physical activity and cause poor sleep quality, which could contribute to obesity, depression, and osteoporosis. All of these comorbidities can increase mortality in patients with asthma (48).

Subgroup analyses showed that male asthma patients have no significantly increased risk of all-cause mortality. We found the negative outcome in male asthma patients mainly because the large-scale study (27) provided a high outlier HR value with small CI. It broadened the CI for the all-cause mortality estimate in male asthma patients so that there was no significant increase. As mentioned above, patients in this large-scale study (27) had active and more severe asthma, which resulted in the high outlier RR value. The other four studies in this subgroup analysis relied on exposure history of asthma, which also includes non-active or mild asthma patients. However, sensitivity analysis showed that this study did not significantly increase the estimate. Therefore, we still concluded that male asthma patients have a 52% increase in all-cause mortality, though this difference was not significant.

### Association Between Asthma and CVD Morbidity and Mortality

We found that asthma patients have a increased risk of CVD morbidity and mortality than people without asthma. Finding an increased risk of CVD morbidity in asthma patients is consistent with a previous meta-analysis (13, 14, 50). The biological mechanism by which asthma causes and aggravates CVD is still unclear. However, some mechanisms have been suggested. First, asthma and CVD share some risk factors, such as smoking and chronic inflammation (7, 51). Asthma can cause inflammation not only in the airway, but also in the whole body (systemic). Prior studies found that colchicine can reduce the incidence rate of CVD to 69%, strongly suggesting that inflammation takes part in the start and progression of CVD (52). Second, asthma patients have an increased risk of chronic airflow obstruction, which can cause lung function decline (53). Impaired lung function is associated with increased levels of inflammatory biomarkers in the circulation (54) and inflammation. Third, asthma medication can cause CVD. Inhaled β2-agonists are associated with an increased risk of adverse cardiovascular outcomes (49).

Subgroup analyses indicate that both male and female asthma patients have a significantly increased risk of CVD morbidity. This outcome is in contrast to a previous meta-analysis (13, 14) that found no significantly increased risk of CVD morbidity in male asthma patients. Our finding confirmed that asthma patients have a significantly increased risk of CVD morbidity, regardless of gender. However, we noted that female asthma patients suffer from a higher increased risk of CVD morbidity than male asthma patients (39 vs. 19%). This gender difference may be associated with sex hormone. Some studies found that estrogen can cause low levels of systemic inflammation by modulating the release of proinflammatory cytokines and regulating the production of leukotrienes. In contrast, androgen may have anti-inflammation effects and protect against airway inflammation (25). As mentioned above, chronic inflammation is one of the risk factors for CVD. Therefore, for women, asthmatic state and estrogen effects can cause increased CVD risk through chronic inflammation.

Subgroup analyses showed that both early and late-onset asthma patients have a significantly increased risk of CVD morbidity. However, late-onset asthma patients have a higher increased risk than early-onset asthma patients (39 vs. 26%). Early and late-onset asthma patients may have different epidemiological characteristics. Most early-onset asthma cases are caused by allergy, whereas late-onset asthma has a complex etiology, such as smoking, occupational, and non-allergic sensitizers, which are related to CVD risk. Moreover, CVD generally occurs among the elderly. Studies of early-onset asthma had difficulties in following participants until their elderly years, which means that their follow-up was relatively insufficient to meet the outcomes of CVD.

## Limitations

The conclusions gained from this study are weakened by the inherent limitations of meta-analyses and observational cohort studies. Although most studies included reported adjusted estimates, the confounding factors of each study were not exactly the same. In addition, some of studies did not adjust for confounding factors. This led to an increased risk of bias in our meta-analysis. Furthermore, most estimates had high heterogeneity, probably because the population characteristics, diagnostic criteria for asthma, and definition of CVD in each study were not completely identical. Finally, all the included data were aggregate, and no individual-level data were available.

## Conclusion

Our study provides evidence that asthma patients have increased risk of all-cause mortality and CVD morbidity and mortality. In addition, subgroup analyses showed that female asthma patients have a higher risk of all-cause mortality and CVD morbidity, and that late-onset asthma patients have higher risk of CVD morbidity. This information reminds clinicians to be aware of the risk of CVD and all-cause mortality in asthma patients.

## Data Availability Statement

The original contributions presented in the study are included in the article/Supplementary Material, further inquiries can be directed to the corresponding author.

## Author Contributions

Z-FL: analysis of data, interpretation results, and drafting and editing the article. BZ, Z-YA, DL, A-JS, QR, and W-BC: design of study, solution of problem, and reviewing the article. LZ, J-YW, M-DH, and Y-JJ: extraction of data and assessment of articles quality. All authors contributed to the article and approved this version of article.

## Funding

The study was financially supported by the Youth Project of Guangdong Provincial Medical Research Fund (Grant No. B2021353), Outstanding Youth Fund Projects of Jiangmen Central Hospital (Grant Nos. J202101 and J202003), the project of Fundamental Research Funds of Jiangmen Central Hospital (Grant No. D201901), and the projects of Jiangmen City Science and Technology Plans (Grant Nos. 2021YLA01033, 2020YLA100, and 2020YLA133).

## Conflict of Interest

The authors declare that the research was conducted in the absence of any commercial or financial relationships that could be construed as a potential conflict of interest.

## Publisher's Note

All claims expressed in this article are solely those of the authors and do not necessarily represent those of their affiliated organizations, or those of the publisher, the editors and the reviewers. Any product that may be evaluated in this article, or claim that may be made by its manufacturer, is not guaranteed or endorsed by the publisher.

## Supplementary Material

Supplementary Figure 1 Funnel plot depicting publication bias for the association between asthma and CVD morbidity. SE, standard error; RR, relative risk.

Supplementary Figure 2 Funnel plot depicting publication bias for the association between asthma and CVD mortality. SE, standard error; RR, relative risk.

Supplementary Figure 3 Funnel plot depicting publication bias for the association between asthma and all-cause mortality. SE, standard error; RR, relative risk.

Supplementary Figure 4 Sensitivity analysis of association between asthma and CVD morbidity. CI, confidential interval; HF, heart failure; CHD, coronary heart disease; a/f, adult female; a/m, adult male; c/f, child female; c/m, child male; int, intermittent; per, persistent.

Supplementary Figure 5 Sensitivity analysis of association between asthma and CVD mortality. CI, confidential interval.

Supplementary Figure 6 Sensitivity analysis of association between asthma and all-cause mortality. CI, confidential interval; int, intermittent; per, persistent.

Supplementary Figure 7 Sensitivity analysis of association between asthma and all-cause mortality in male patients. CI, confidential interval.

Supplementary Figure 8 Sensitivity analysis of association between asthma and all-cause mortality in female patients. CI, confidential interval.

Supplementary Figure 9 Sensitivity analysis of association between asthma and CVD morbidity in early-onset patients. CI, confidential interval; HF, heart failure; CHD, coronary heart disease; a/f, adult female; a/m, adult male; c/f, child female; c/m, child male.

Supplementary Figure 10 Sensitivity analysis of association between asthma and CVD morbidity in late-onset patients. CI, confidential interval; HF, heart failure; CHD, coronary heart disease; a/f, adult female; a/m, adult male; c/f, child female; c/m, child male.

Supplementary Figure 11 Sensitivity analysis of association between asthma and CVD morbidity in male patients. CI, confidential interval; a/f, adult female; a/m, adult male; c/f, child female; c/m, child mal.

Supplementary Figure 12 Sensitivity analysis of association between asthma and CVD morbidity in female patients. CI, confidential interval; a/f, adult female; a/m, adult male; c/f, child female; c/m, child male.

Supplementary Figure 13 Sensitivity analysis of association between asthma and all-cause mortality by omitting two outliers. CI, confidential interval; int, intermittent; per, persistent.

Supplementary Table 1 Search strategies.

Supplementary Table 2 Newcastle-ottawa scale for assessing the quality of included studies.

Supplementary Table 3 PRISMA 2009 checklist.

## Abbreviations

CVD: cardiovascular disease
CI: confidential interval
RR: relative risk
HR: hazard ratio
NOS: Newcastle-Ottawa Scale
PRISMA: Preferred Reporting Items for Systematic Reviews and Meta-Analyses
MOOSE: Meta-Analysis of Observational Studies in Epidemiology
PROSPERO: Prospective Register of Systematic Reviews.
