# Supplementary figures and images for: Association Between Asthma and All-Cause Mortality and Cardiovascular Disease Morbidity and Mortality: A Meta-Analysis of Cohort Studies
Source: Front Cardiovasc Med. 2022 Mar 17;9:861798. doi: 10.3389/fcvm.2022.861798 (PMC8968068; doi:10.3389/fcvm.2022.861798)

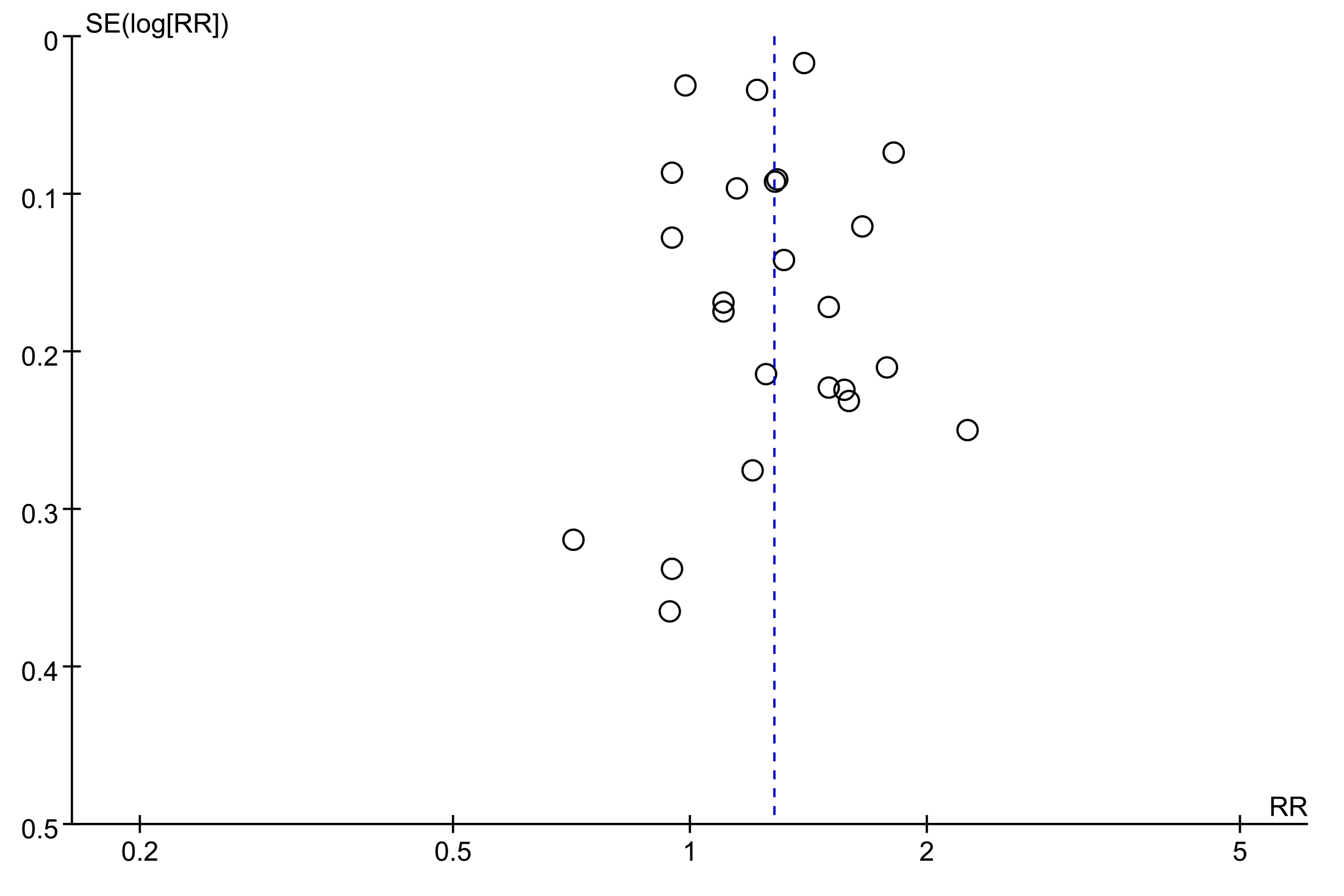

Supplement: Supplementary Figure 1 — Funnel plot depicting publication bias for the association between asthma and CVD morbidity. SE, standard error; RR, relative risk. [file Image_1.TIF]

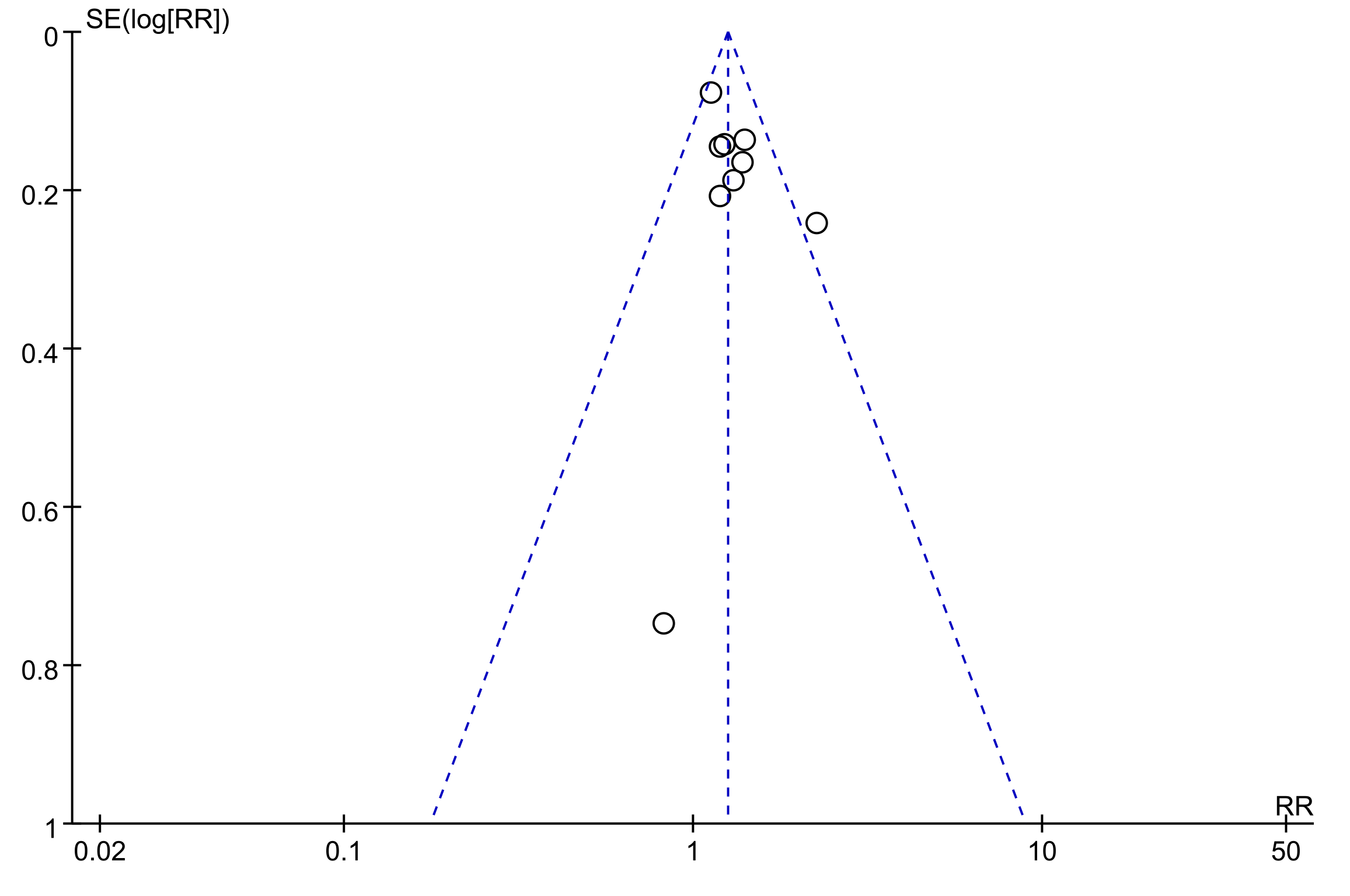

Supplement: Supplementary Figure 2 — Funnel plot depicting publication bias for the association between asthma and CVD mortality. SE, standard error; RR, relative risk. [file Image_2.TIF]

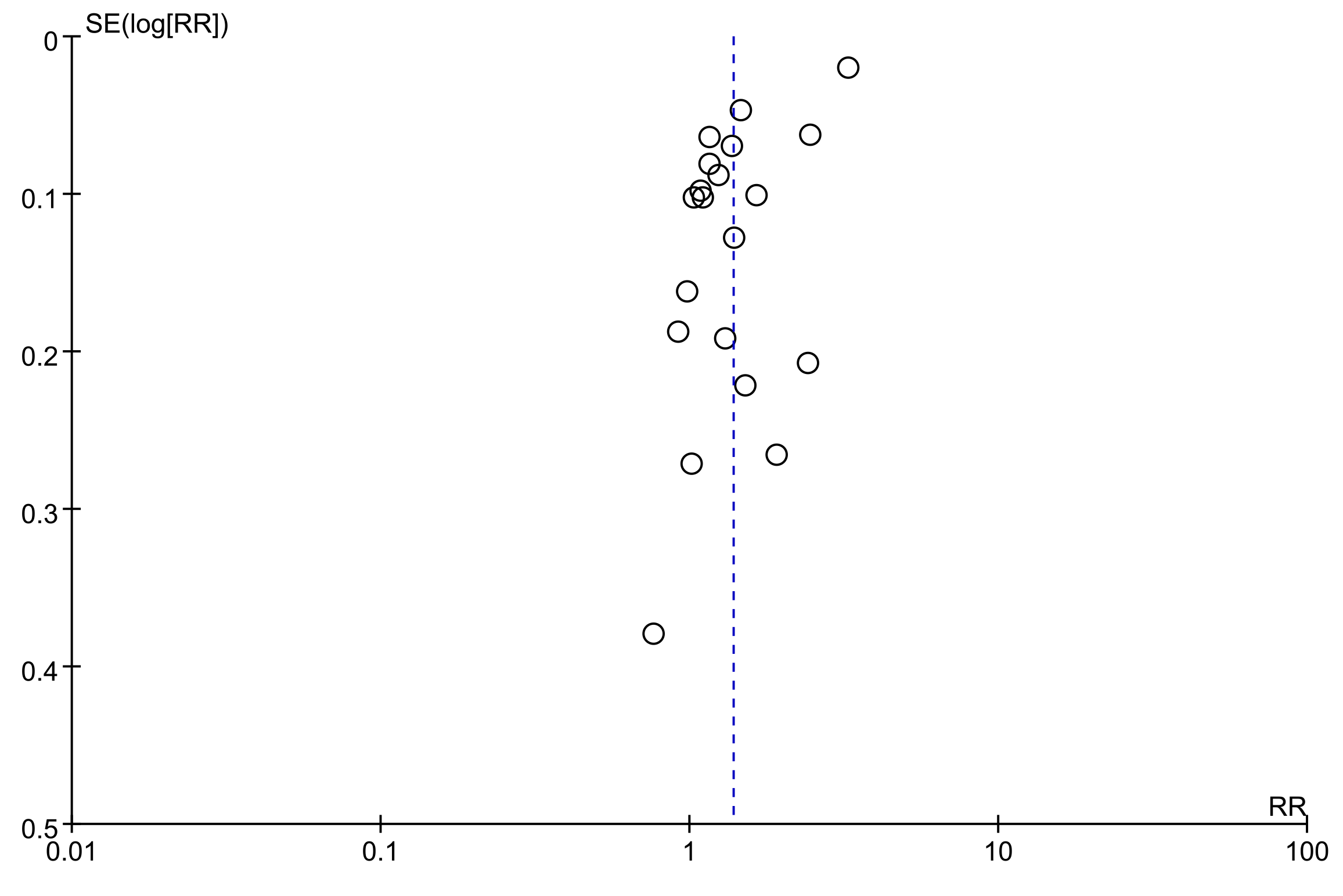

Supplement: Supplementary Figure 3 — Funnel plot depicting publication bias for the association between asthma and all-cause mortality. SE, standard error; RR, relative risk. [file Image_3.TIF]

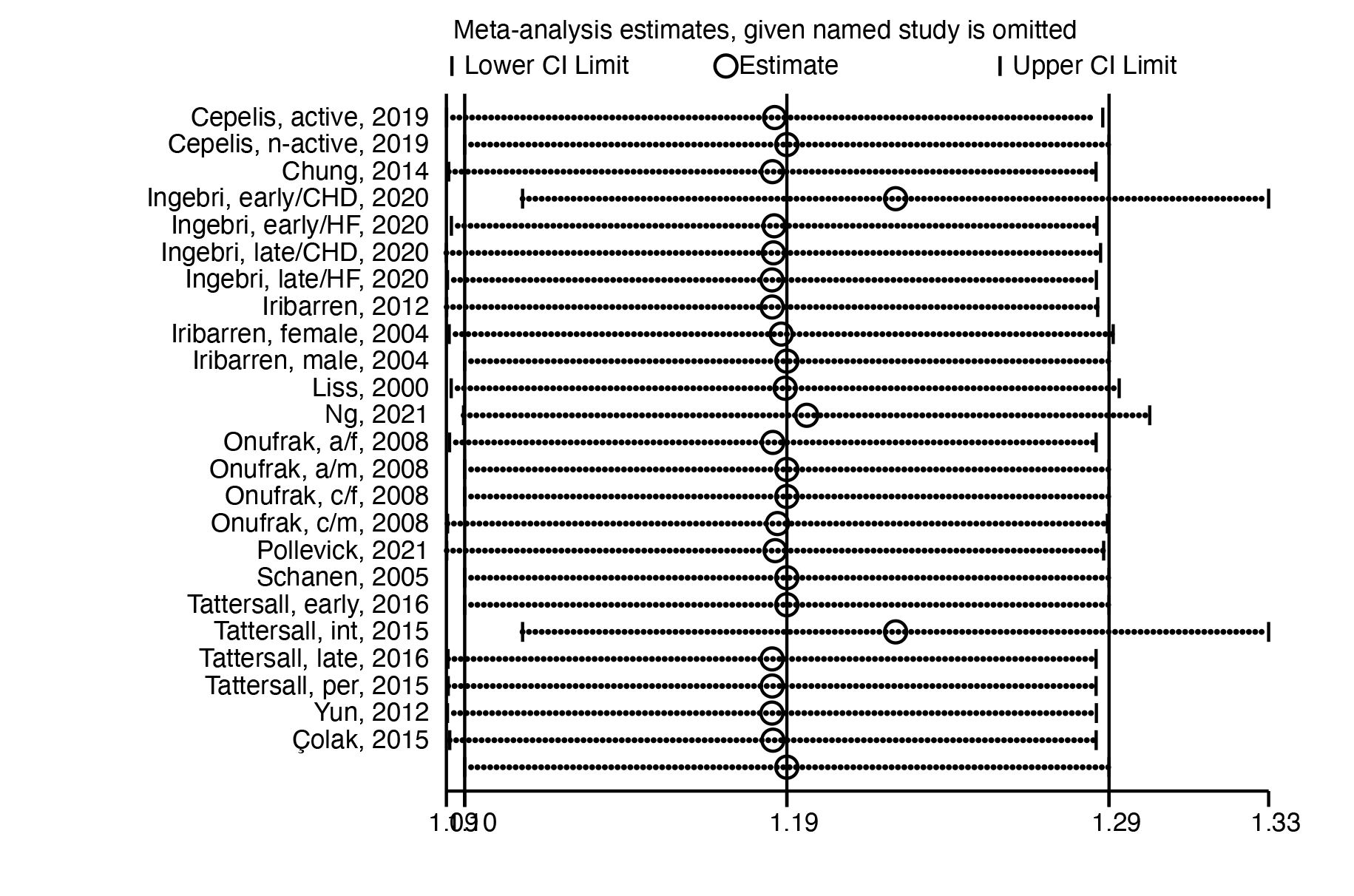

Supplement: Supplementary Figure 4 — Sensitivity analysis of association between asthma and CVD morbidity. CI, confidential interval; HF, heart failure; CHD, coronary heart disease; a/f, adult female; a/m, adult male; c/f, child female; c/m, child male; int, intermittent; per, persistent. [file Image_4.TIF]

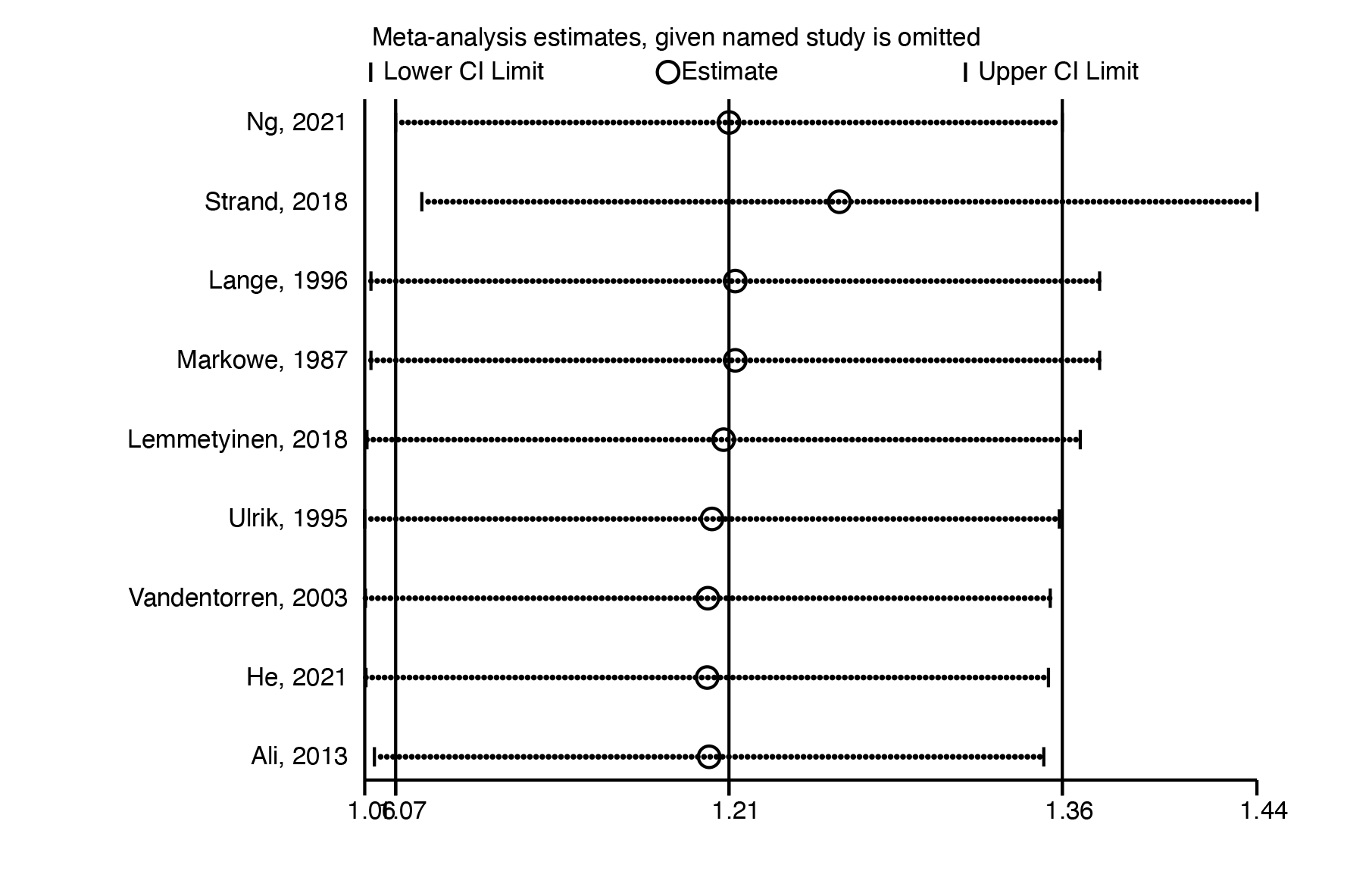

Supplement: Supplementary Figure 5 — Sensitivity analysis of association between asthma and CVD mortality. CI, confidential interval. [file Image_5.TIF]

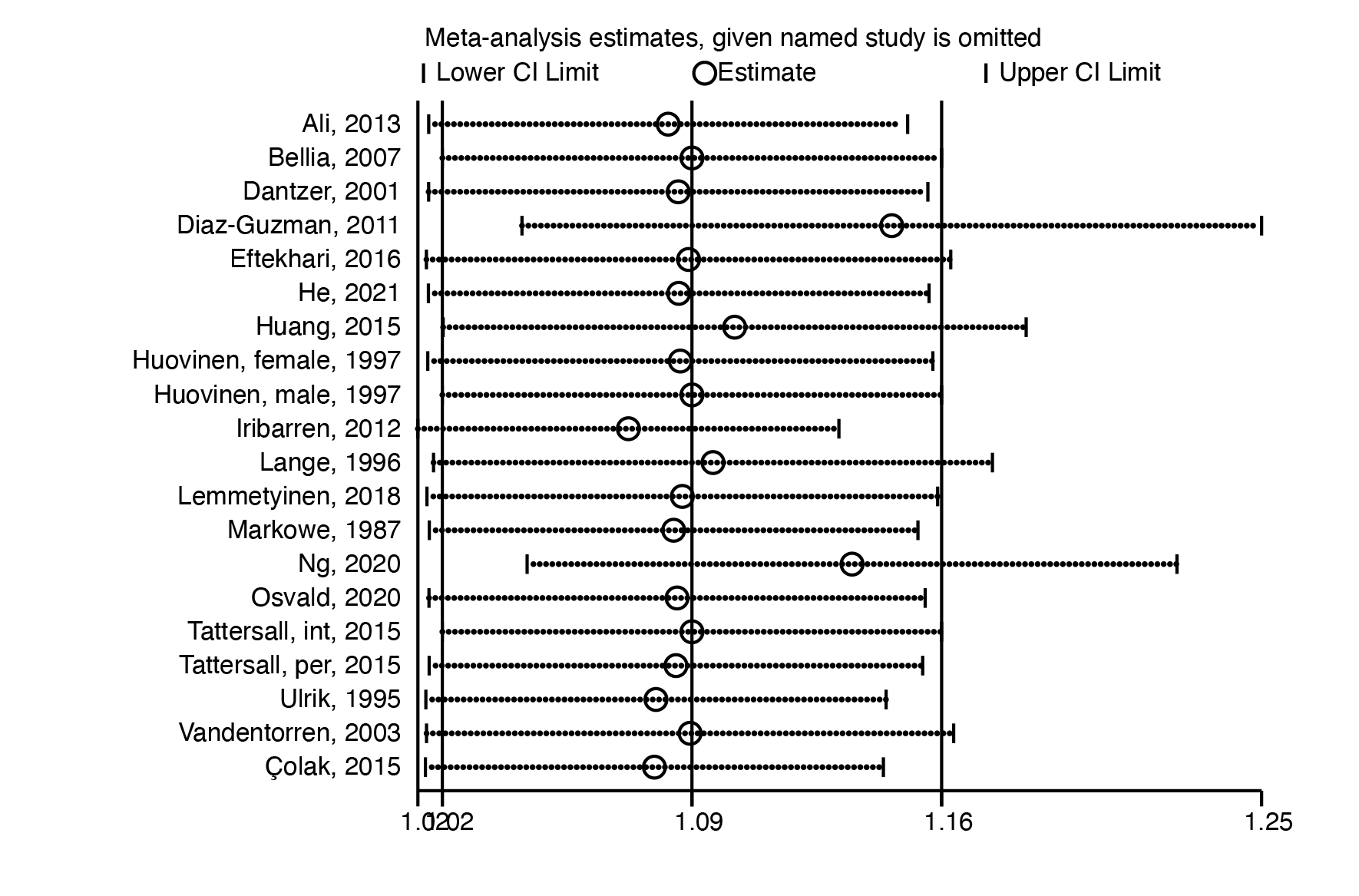

Supplement: Supplementary Figure 6 — Sensitivity analysis of association between asthma and all-cause mortality. CI, confidential interval; int, intermittent; per, persistent. [file Image_6.TIF]

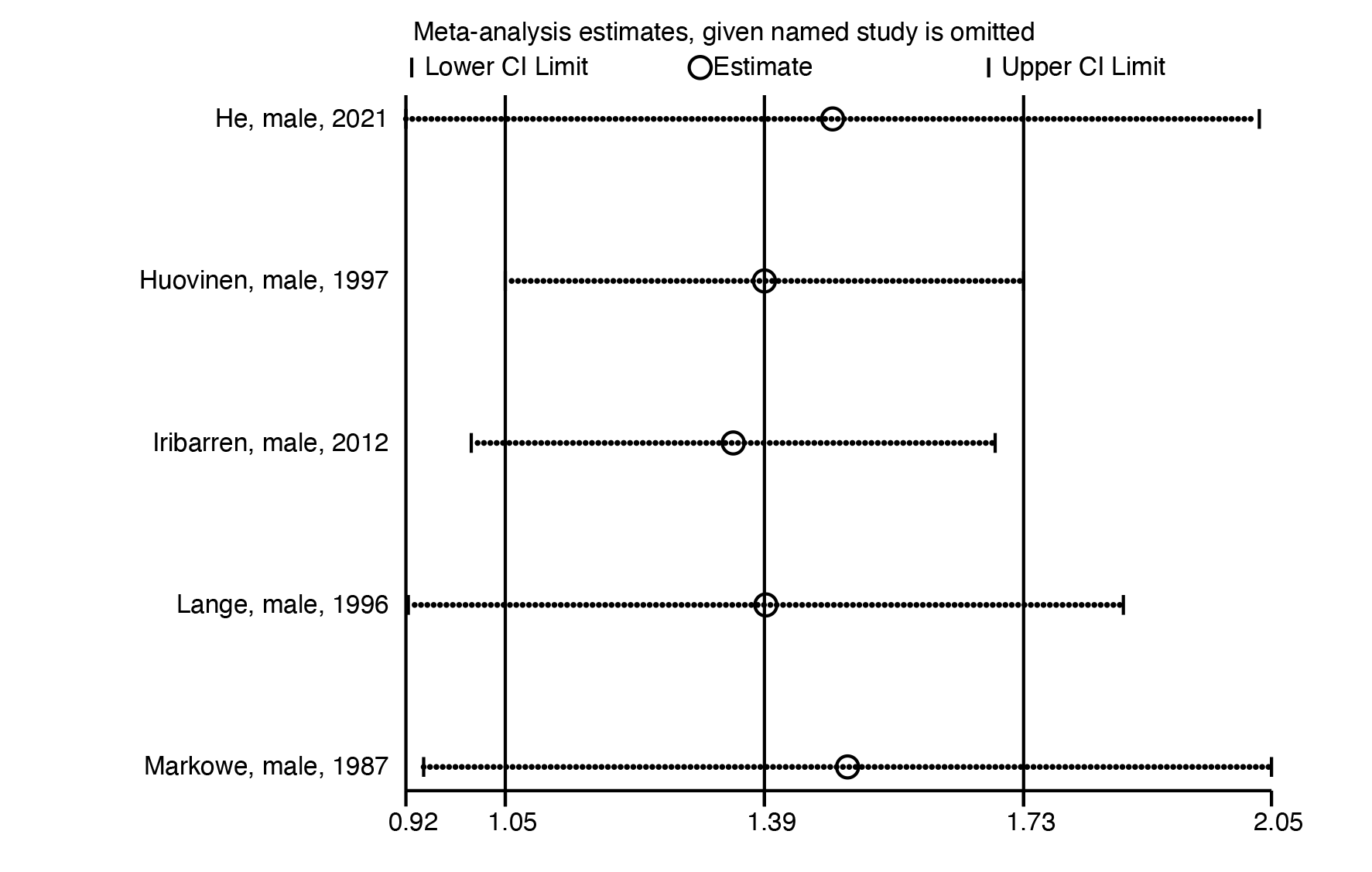

Supplement: Supplementary Figure 7 — Sensitivity analysis of association between asthma and all-cause mortality in male patients. CI, confidential interval. [file Image_7.TIF]

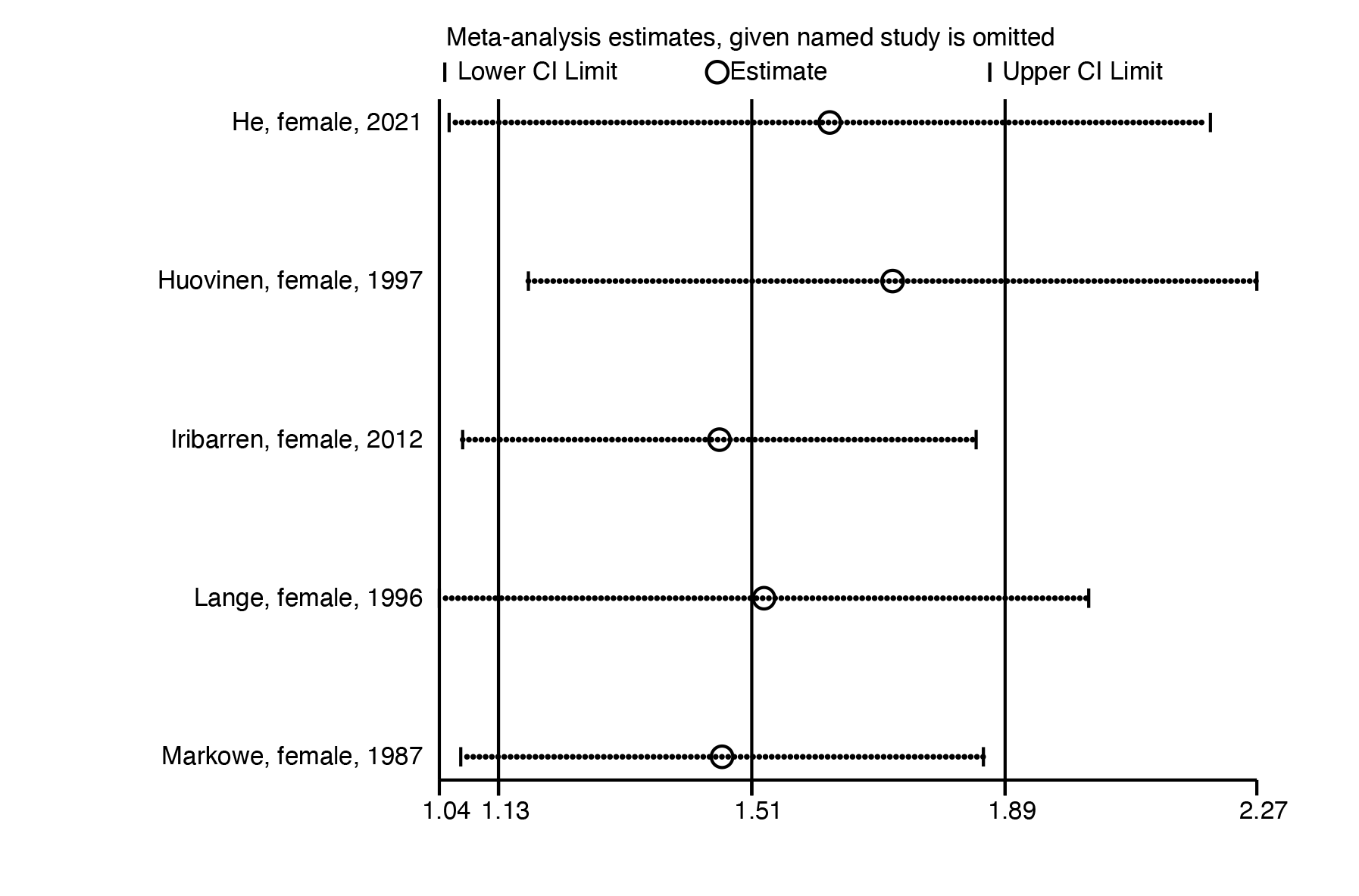

Supplement: Supplementary Figure 8 — Sensitivity analysis of association between asthma and all-cause mortality in female patients. CI, confidential interval. [file Image_8.TIF]

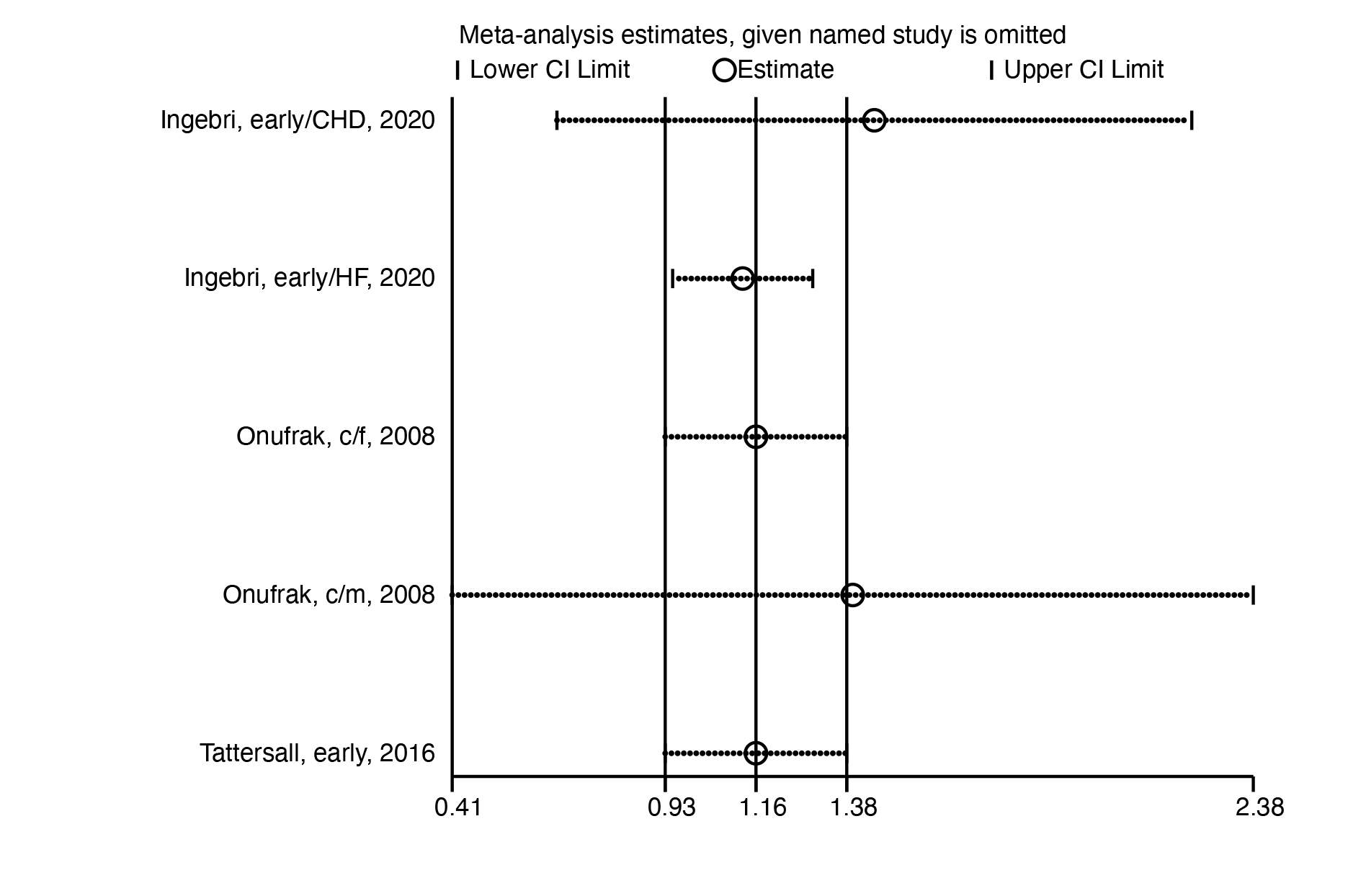

Supplement: Supplementary Figure 9 — Sensitivity analysis of association between asthma and CVD morbidity in early-onset patients. CI, confidential interval; HF, heart failure; CHD, coronary heart disease; a/f, adult female; a/m, adult male; c/f, child female; c/m, child male. [file Image_9.TIF]

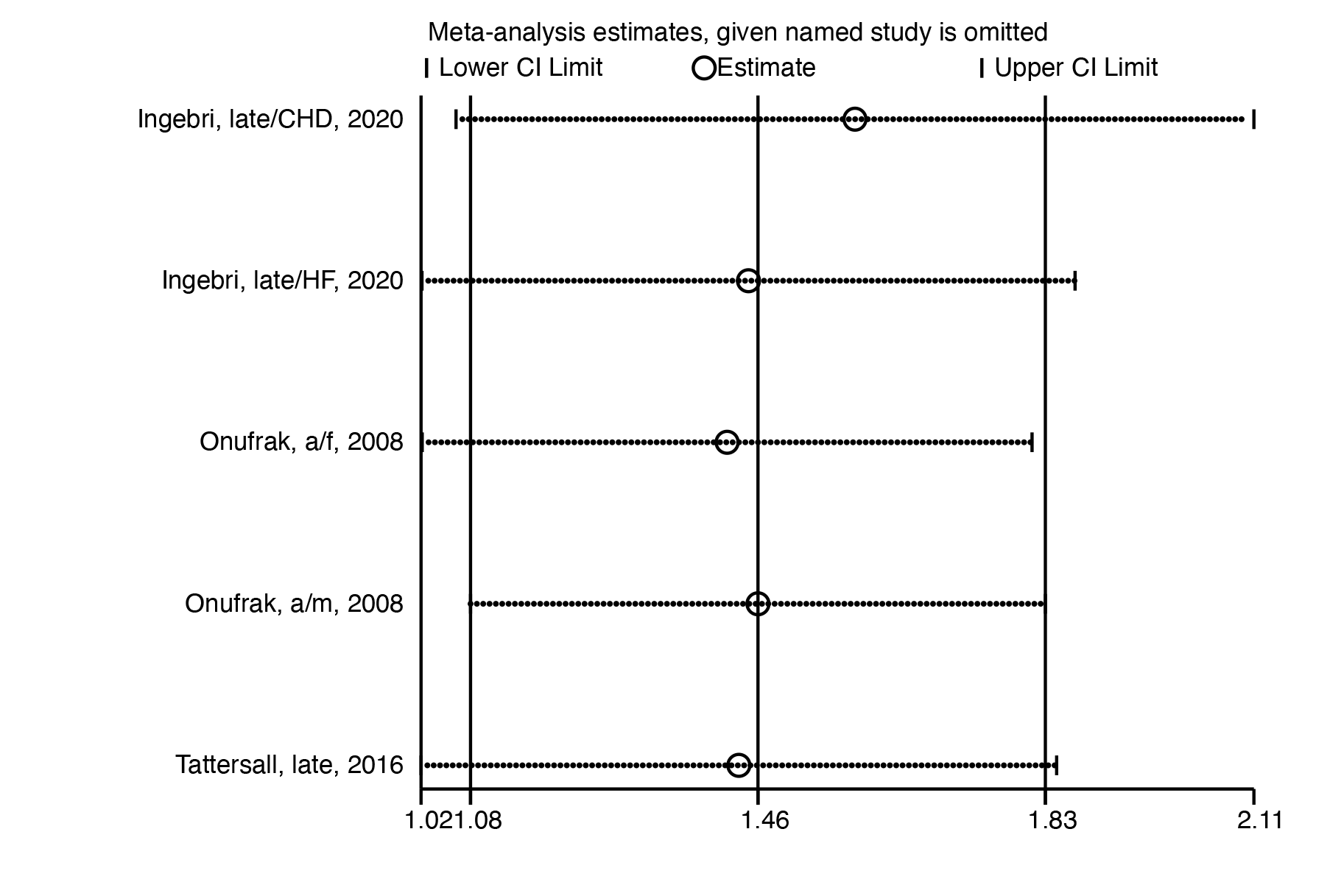

Supplement: Supplementary Figure 10 — Sensitivity analysis of association between asthma and CVD morbidity in late-onset patients. CI, confidential interval; HF, heart failure; CHD, coronary heart disease; a/f, adult female; a/m, adult male; c/f, child female; c/m, child male. [file Image_10.TIF]

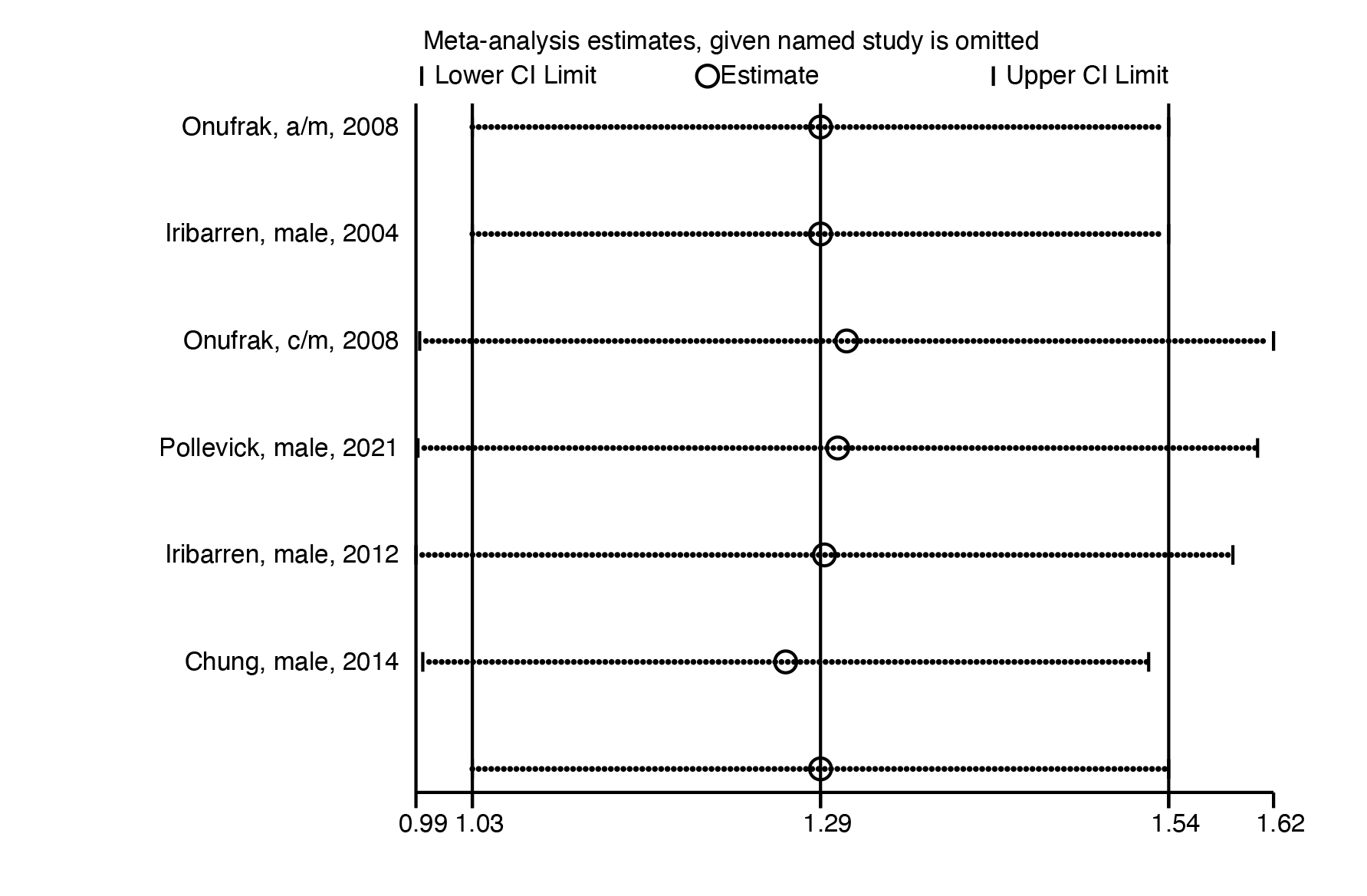

Supplement: Supplementary Figure 11 — Sensitivity analysis of association between asthma and CVD morbidity in male patients. CI, confidential interval; a/f, adult female; a/m, adult male; c/f, child female; c/m, child mal. [file Image_11.TIF]

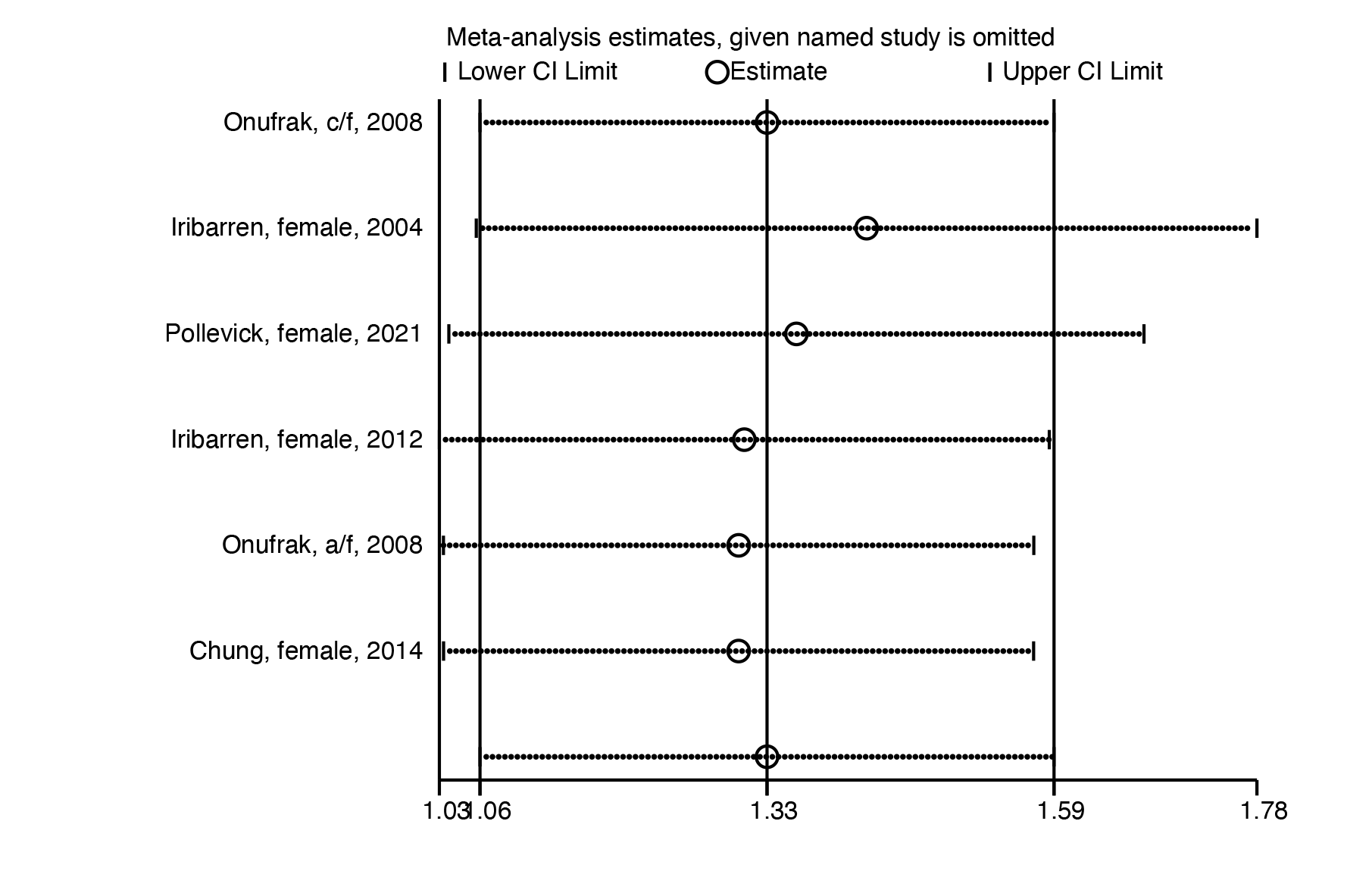

Supplement: Supplementary Figure 12 — Sensitivity analysis of association between asthma and CVD morbidity in female patients. CI, confidential interval; a/f, adult female; a/m, adult male; c/f, child female; c/m, child male. [file Image_12.TIF]

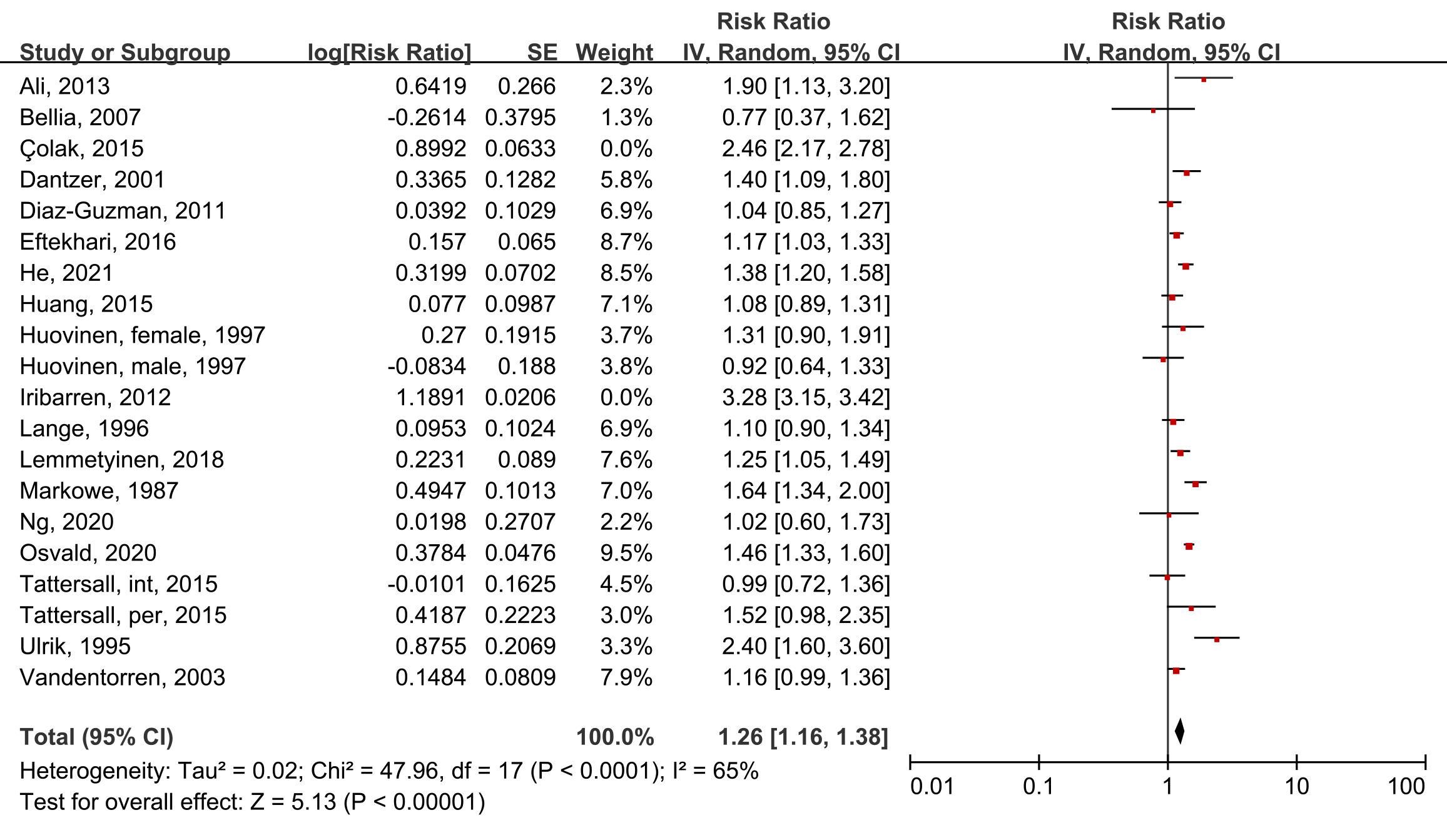

Supplement: Supplementary Figure 13 — Sensitivity analysis of association between asthma and all-cause mortality by omitting two outliers. CI, confidential interval; int, intermittent; per, persistent. [file Image_13.TIF]
