# Supplementary material for: Association Between Asthma and All-Cause Mortality and Cardiovascular Disease Morbidity and Mortality: A Meta-Analysis of Cohort Studies
Source: Front Cardiovasc Med. 2022 Mar 17;9:861798. doi: 10.3389/fcvm.2022.861798 (PMC8968068; doi:10.3389/fcvm.2022.861798)
Supplement: Supplementary Table 1 — Search strategies. [file Table_1.DOCX]

| Table E1 | |
| --- | --- |
| Search Strategies | |
| PubMed |  |
| Search number | Query |
| #1 | cardiovascular disease*[title] |
| #2 | coronary disease*[title] |
| #3 | Myocardial Ischemia[title] |
| #4 | coronary artery disease*[title] |
| #5 | myocardial infarction[title] |
| #6 | cardiovascular diseases[Mesh] |
| #7 | coronary disease[Mesh] |
| #8 | Myocardial Ischemia[Mesh] |
| #9 | coronary artery disease[Mesh] |
| #10 | myocardial infarction[Mesh] |
| #11 | #1 OR #2 OR #3 OR #4 OR #5 OR #6 OR #7 OR #8 OR #9 OR #10 |
| #12 | Mortality[title] |
| #13 | Death[title] |
| #14 | fatal[title] |
| #15 | Survival[title] |
| #16 | Mortality[Subheading] |
| #17 | mortality[Mesh] |
| #18 | death[Mesh] |
| #19 | #12 OR #13 OR #14 OR #15 OR #16 OR #17 OR #18 |
| #20 | #11 OR #19 |
| #21 | epidemiologic studies[MeSH] |
| #22 | cohort studies[MeSH] |
| #23 | epidemiologic[Text Word] |
| #24 | longitudinal[Text Word] |
| #25 | cohort[Text Word] |
| #26 | follow up[Text Word] |
| #27 | observational[Text Word] |
| #28 | prospective[Text Word] |
| #29 | retrospective[Text Word] |
| #30 | #21 OR #22 OR #23 OR #24 OR #25 OR #26 OR #27 OR #28 OR #29 |
| #31 | #20 AND #30 |
| #32 | asthma*[title] |
| #33 | allergic airway inflammation[title] |
| #34 | #32 OR #33 |
| #35 | #31 AND #34 |
| #36 | review[Publication Type] |
| #37 | #35 NOT #36 |
| #38 | COVID[title/abstract] |
| #39 | Coronaviruses[MeSH] |
| #40 | Coronaviruses[Title/Abstract] |
| #41 | 2019-nCoV[Title/Abstract] |
| #42 | SARS-CoV-2[Title/Abstract] |
| #43 | #38 OR #39 OR #40 OR #41 OR #42 |
| #44 | #37 NOT #43 |
| Embase |  |
| #1 | ‘cardiovascular disease*’:ti |
| #2 | ‘coronary disease*’:ti |
| #3 | ‘myocardial Ischemia’:ti |
| #4 | ‘coronary artery disease*’:ti |
| #5 | ‘myocardial infarction’:ti |
| #6 | ‘cardiovascular diseases’/exp |
| #7 | ‘coronary artery disease’/exp |
| #8 | ‘heart muscle ischemia’/exp |
| #9 | ‘heart infraction’/exp |
| #10 | #1 OR #2 OR #3 OR #4 OR #5 OR #6 OR #7 OR #8 OR #9 |
| #11 | mortality:ti |
| #12 | death:ti |
| #13 | survival:ti |
| #14 | fatal:ti |
| #15 | ‘mortality’/exp |
| #16 | #11 OR #12 OR #13 OR #14 OR #15 |
| #17 | #10 OR #16 |
| #18 | ‘cohort analysis’/exp |
| #19 | cohort |
| #20 | ‘retrospective’:ti |
| #21 | ‘prospective’:ti |
| #22 | ‘observational’:ti |
| #23 | #18 OR #19 OR #20 OR #21 OR #22 |
| #24 | #17 AND #23 |
| #25 | asthma:ti |
| #26 | ‘allergic airway inflammation’:ti |
| #27 | #25 OR #26 |
| #28 | #24 AND #27 |
| #29 | review:ti |
| #30 | #28 NOT #29 |
| #31 | [article]/lim |
| #32 | #30 AND #31 |
| #33 | [humans]/lim |
| #34 | #32 AND #33 |
