# Supplementary material for: Association Between Asthma and All-Cause Mortality and Cardiovascular Disease Morbidity and Mortality: A Meta-Analysis of Cohort Studies
Source: Front Cardiovasc Med. 2022 Mar 17;9:861798. doi: 10.3389/fcvm.2022.861798 (PMC8968068; doi:10.3389/fcvm.2022.861798)
Supplement: Supplementary Table 2 — Newcastle-ottawa scale for assessing the quality of included studies. [file Table_2.DOCX]

| Table E2 | | | | |
| --- | --- | --- | --- | --- |
| Newcastle-Ottawa Scale for Assessing the Quality of Included Studies | | | | |
| Author, Year | **Selection (max=4)** | **Comparability (max=2)** | **Exposure/Outcome (max=3)** | **Overall quality score (max=9)** |
| Ingebri et al, 2020 | 4 | 2 | 2 | 8 |
| Bellia et al, 2007 | 3 | 2 | 3 | 8 |
| Liss et al, 2000 | 2 | 1 | 3 | 6 |
| Çolak et al, 2015 | 3 | 2 | 2 | 7 |
| Lange et al，1996 | 4 | 2 | 2 | 8 |
| Ali et al, 2013 | 4 | 1 | 3 | 8 |
| Dantzer et al, 2001 | 3 | 1 | 3 | 7 |
| Diaz-Guzman et al, 2011 | 2 | 2 | 3 | 7 |
| Huovinen et al, 1997 | 4 | 2 | 3 | 9 |
| Markowe et al, 1987 | 3 | 2 | 3 | 8 |
| Ulrik et al, 1995 | 3 | 2 | 3 | 8 |
| Vandentorren et al, 2003 | 3 | 2 | 3 | 8 |
| Chung et al, 2014 | 4 | 2 | 2 | 8 |
| Iribarren et al, 2004 | 4 | 1 | 2 | 7 |
| Iribarren et al, 2012 | 3 | 2 | 2 | 7 |
| Onufrak et al, 2008 | 3 | 2 | 3 | 8 |
| Schanen et al, 2005 | 3 | 2 | 2 | 7 |
| Yun et al, 2012 | 3 | 2 | 2 | 7 |
| Ng, et al, 2020 | 3 | 2 | 2 | 7 |
| Eftekhari, et al, 2016 | 3 | 2 | 3 | 8 |
| Strand, et al, 2018 | 2 | 2 | 3 | 7 |
| Lemmetyinen et al, 2018 | 3 | 2 | 3 | 8 |
| Cepelis et al, 2019 | 3 | 2 | 3 | 8 |
| Caffrey et al, 2020 | 4 | 1 | 3 | 8 |
| He et al, 2021 | 3 | 1 | 3 | 7 |
| Huang et al, 2015 | 4 | 1 | 3 | 8 |
| Pollevick et al, 2021 | 4 | 1 | 3 | 8 |
| Ng et al, 2021 | 3 | 1 | 3 | 7 |
| Tattersall et al, 2016 | 3 | 1 | 3 | 7 |
| Tattersall et al, 2015 | 4 | 1 | 3 | 8 |
